# Isolation of a bacteriophage targeting *Pseudomonas aeruginosa* and exhibits a promising in vivo efficacy

**DOI:** 10.1186/s13568-023-01582-3

**Published:** 2023-07-26

**Authors:** Aliaa Abdelghafar, Amira El-Ganiny, Ghada Shaker, Momen Askoura

**Affiliations:** Department of Microbiology and Immunology, Faculty of Pharmacy, Zagazig University, Zagazig, 44519 Egypt

**Keywords:** Antibiotic resistance, Bacteriophage, Biofilm, Genomic analysis, Phage therapy, *Pseudomonas aeruginosa*

## Abstract

*Pseudomonas aeruginosa* is an important pathogen that causes serious infections. Bacterial biofilms are highly resistant and render bacterial treatment very difficult, therefore necessitates alternative antibacterial strategies. Phage therapy has been recently regarded as a potential therapeutic option for treatment of bacterial infections. In the current study, a novel podovirus vB_PaeP_PS28 has been isolated from sewage with higher lytic activity against *P. aeruginosa*. Isolated phage exhibits a short latent period, large burst size and higher stability over a wide range of temperatures and pH. The genome of vB_PaeP_PS28 consists of 72,283 bp circular double-stranded DNA, with G + C content of 54.75%. The phage genome contains 94 open reading frames (ORFs); 32 for known functional proteins and 62 for hypothetical proteins and no tRNA genes. The phage vB_PaeP_PS28 effectively inhibited the growth of *P. aeruginosa* planktonic cells and displayed a higher biofilm degrading capability. Moreover, therapeutic efficacy of isolated phage was evaluated in vivo using mice infection model. Interestingly, survival of mice infected with *P. aeruginosa* was significantly enhanced upon treatment with vB_PaeP_PS28. Furthermore, the bacterial load in liver and kidney isolated from mice infected with *P. aeruginosa* and treated with phage markedly decreased as compared with phage-untreated *P. aeruginosa*-infected mice. These findings support the efficacy of isolated phage vB_PaeP_PS28 in reducing *P. aeruginosa* colonization and pathogenesis in host. Importantly, the isolated phage vB_PaeP_PS28 could be applied alone or as combination therapy with other lytic phages as phage cocktail therapy or with antibiotics to limit infections caused by *P. aeruginosa*.

**Supplementary Information:**

The online version contains supplementary material available at 10.1186/s13568-023-01582-3.

## Introduction

*Pseudomonas aeruginosa* is a causative agent of wide variety of infections ranging from soft tissue infections to life-threatening ones including bacteremia and pneumonia. Furthermore, *Pseudomonas aeruginosa* is a global opportunistic pathogen and a major cause of nosocomial infections due to the flexibility and adaptability encoded in its genome (Gellatly and Hancock 2013). *P. aeruginosa* could grow on different medical equipment due to the presence of its essential binding factors such as flagella, pili and the ability to form biofilms (Remold et al. 2011; Gale et al. 2015). Unfortunately, infections caused by this bacterium are characterized by higher morbidity and mortality rates especially in immunocompromised patients and those suffering from severe burns or cystic fibrosis (Guarner and Malagelada 2003; English and Gaur 2010).

Numerous virulence factors contribute to *P. aeruginosa* pathogenesis including hemolysins, rhamnolipids, proteases and biofilms (Lee and Zhang 2015). *P. aeruginosa* is capable of forming biofilms which protect bacteria from environmental stresses and phagocytosis and lead to long-term persistence (Moradali et al. 2017). Moreover, *P. aeruginosa* is able to acquire resistance through mutation and horizontal gene transfer added to its intrinsic resistance to various antibiotics including beta-lactams (Fajardo et al. 2008; Breidenstein et al. 2011). Therefore, treatment of *P. aeruginosa* infections by conventional antibiotics has become a major global challenge due to bacterial resistance.

Recently, due to increased antimicrobial resistance, there is an urgent necessity for the development of alternative antimicrobial approaches to efficiently control bacterial infections (Cegelski et al. 2008). Bacteriophages (phages) have been considered as a potential therapeutic option due to their safety and avoidance of harm to normal flora (Taati Moghadam et al. 2020). Meanwhile, antibiotic resistance increases, phages retain its ability to compact antibiotic-resistant bacteria in addition to its ability to inhibit biofilms (Abedon et al. 2017; Taati et al. 2020). Furthermore, there are no obvious adverse effects associated with phage therapy due to the higher phage specificity towards target bacterial species without affecting the host microbiota (Skurnik et al. 2007). Phages have valuable properties such as they are easy to culture, economic and can be stored for long periods (Pohane et al. 2014). Furthermore, endotoxin released upon lysis of bacterial cells following treatment with phages is relatively lower compared to that released by antibiotics (Dufour et al. 2017). Therefore, phage therapy is believed to be a promising tool for management of bacterial infections and therefore could be considered for treatment of *P. aeruginosa* infections alone or in combination with antibiotics (Krylov et al. 2015; Thanh et al. 2020).

While a lot of previous studies have isolated several phages infecting *P. aeruginosa*, many of these studies suffer from serious gaps regarding detailed characterization of isolated phages. For instance, the genomes of some previously isolated phages have not been fully characterized (Kumari et al. 2009; Azizian et al. 2015; Didamony et al. 2015; Barazandeh et al. 2021). Furthermore, neither the antibiofilm potential of isolated phages nor their in vivo antibacterial efficacy has been investigated in these studies (Miyata et al. 2014; Cao et al. 2015; Shigehisa et al. 2016; Tang et al. 2018; de Melo et al. 2019; Alvi et al. 2020; Enwuru et al. 2021; Namonyo et al. 2022). Therefore, complete genome analysis, the antibiofilm activity and the ability of isolated phage to reduce *P. aeruginosa* pathogenesis in vivo will be fully covered herein. The current study aims to isolate and characterize a virulent phage targeting *P. aeruginosa* isolated from various clinical sources. The physical properties, antibiofilm activity as well as whole genome sequencing of isolated phage will be determined herein. Moreover, the influence of isolated phage on *P. aeruginosa* pathogenesis in host will be assessed in vivo using mice infection model. The findings of present study would be of great importance and helpful in treatment of *P. aeruginosa* related infections.

## Material and methods

### Isolation and identification of *P. aeruginosa*

Clinical *P. aeruginosa* isolates were provided by the clinical laboratories of Zagazig University Hospitals, Zagazig, Egypt with no direct involvement of patients in the study. *P. aeruginosa* isolates were further identified biochemically according to Douraghi et al. (2014). In addition to clinical isolates, *P. aeruginosa* reference strains; ATCC 27853, ATCC 9027 and PAO1 were included in this study. Bacterial strains were stored in Muller Hinton (MH) broth containing 20% glycerol and kept at − 80 °C. The bacterial strains used in current study are listed in **(**Additional file 1: Table S1).

### Antimicrobial susceptibility testing

The susceptibility of *P. aeruginosa* strains to different antibiotics was determined by Kirby-Bauer standard disc diffusion method (Patel et al. 2015). Diameters of inhibition zones were measured and bacterial susceptibility to antibiotics was interpreted as resistant (R), intermediate (I) and susceptible (S) according to guidelines recommended by CLSI, (2018) (Humphries et al. 2018).

### Quantitative assessment of biofilm formation

The capacity of *P. aeruginosa* to form biofilm was assayed spectrophotometrically as previously reported by Stepanović et al. (2007). In brief, bacterial suspensions were allowed to form biofilms in 96-well polystyrene U-shaped microtiter plate (Costar^™^; Corning^™^) and incubated for 24 h at 37 °C. Wells contained tryptone soya (TS) broth only were included as negative control. Fixed biofilms were stained by using 1% crystal violet and bound dye was dissolved by 33% glacial acetic acid. The optical densities were measured spectrophotometrically at 570 nm (Bio-Tek synergy HT microplate reader, USA). The cut-off optical density (ODc) was calculated as three times standard deviations above the mean OD of the negative control. Bacterial isolates were categorized based on biofilm forming capacity following the criteria mentioned before (Stepanović et al. 2007).

### Bacteriophage isolation

The phage was isolated from sewage by the enrichment technique (Didamony et al. 2015; Chen et al. 2021). The sample was clarified through centrifugation at 6000×*g* for 20 min and filtered through a 0.45 μm membrane filter (Millipore, USA). The filtrate was added to an equal volume of double concentrated TS broth medium containing exponential phase culture of *P. aeruginosa* (PS28) as a host strain. This bacterial strain was isolated from patient suffering from urinary tract infection. Suspensions were incubated in shaker incubator at 37 ºC for 24 h. The cultures were centrifuged at 6000×*g* for 10 min and the supernatants were filtered through 0.22 μm membrane filter to remove bacteria. The filtrates were checked for presence of bacteriophages by the spot assay. Furthermore, the phage presence was characterized by the double agar layer method as described before (Mazzocco et al. 2009). Briefly, a mixture composed of 3 mL of prewarmed soft TS agar (0.6% agar) and 100 µL of *P. aeruginosa* culture grown to the exponential phase were poured over solid bottom TS agar plate. After solidifying, 10 μL of filtered suspension was spotted onto bacterial lawns then left to dry and incubated overnight at 37 °C. Appearance of clear zone (plaques) in the plate indicates presence of bacteriophages.

### Bacteriophage purification and propagation

Bacteriophage purification was done by picking a well isolated single plaque and resuspended in SM buffer [100 mM NaCl, 8 mM MgSO4, 50 mM Tris–HCl (pH 7.4), 0.01% gelatin]. Then, aliquots of 100 μL of serial diluted phage was mixed with 100 μL of *P. aeruginosa* culture (PS28) and plated by soft agar overlay technique. This process was performed successive rounds in order to obtain uniform plaque morphology. Phage propagation was done by incubating the phage with *P. aeruginosa* (PS28) as host with shaking at 120 rpm for 24 h. The culture was centrifuged and the supernatant was filtered. The phage was propagated to obtain a high titer stock as described (Kumari et al. 2009) and purified phage stock was stored at 4 °C (Russell and Sambrook 2001).

### Transmission electron microscopy (TEM)

The phage morphology was visualized using the transmission electron microscope (TEM) as described (Shen et al. 2016). A drop of high titer purified phage [10^12^ particle forming unit (PFU)/mL] was applied to carbon-coated copper grid (200 mesh). Phage particles were negatively stained with 2% phosphotungstic acid (pH 7). Finally, the grid was air-dried and phage particles were examined using TEM (Hitachi H600A, Japan).

### Determination of phage host range and efficiency of plating (EOP)

The host range of isolated phage against a total of 18 *P. aeruginosa* strains (15 *P. aeruginosa* isolates from different clinical sources and 3 *P. aeruginosa* reference strains; *P. aeruginosa* ATCC 27853, *P. aeruginosa* ATCC 9027 and *P. aeruginosa* PAO1) in addition to other bacterial species (*Escherichia coli*, *Salmonella* Typhimurium, *Klebsiella pneumoniae*, *Serratia marcescens* and *Staphylococcus aureus*) was performed using the spot testing method and as described above. The selected clinical *P. aeruginosa* isolates were chosen to be representative for different clinical sources including; burn and urine (4 isolates each), wound and endotracheal aspirates (3 isolates each) and ear infections (1 isolate) in order to give a full picture about susceptibility of *P. aeruginosa* isolates from various clinical sources to infection with isolated phage. Clear inhibition zone was considered as evidence for bacterial susceptibility to phage (Adnan et al. 2020). The efficiency of plating (EOP) of isolated phage was evaluated against *P. aeruginosa* isolates that showed lysis in the spot assay. Aliqutoes of 100 µL of bacterial cultures grown to the exponential phase were co-cultured with 100 µL of tenfold serially diluted phage in soft agar layer and overlayed on surface of TS agar plates. The plates were incubated overnight at 37 °C and the PFUs were counted for each phage-bacterium combination. The EOP values were estimated by dividing the total number of PFUs obtained by the target bacteria to the total number of PFUs obtained by host bacteria; *P. aeruginosa* PS28. Assays were repeated three times and results were recorded according as the follow; High production if the EOP ratio was ≥ 0.5; Medium production if 0.5 > EOP ratio ≥ 0.1; Low production if 0.1 > EOP > 0.001 and inefficient if EOP ≤ 0.001 (Khan Mirzaei and Nilsson 2015).

### Temperature and pH stability

For thermal stability evaluation, aliquot of 100 µL of purified phage particles was mixed with 900 µL of SM buffer and placed in an adjusted water bath incubator at various temperatures (4, 40, 50, 60, 70, 80, 90 and 100 °C) for 1 h. The phage titer was determined after incubation at each specified temperature by the double layer agar technique. Similarly, the impact of pH on phage survival was assessed. Purified phage particles were incubated for 1 h in SM buffer at different pH (3, 4, 5, 6, 7, 8, 9, 10, 11 and 12) adjusted using either 1 M HCL or 1 M NaOH followed by determining the phage titer. These assays were carried out in triplicate and the phage titers were estimated as described before (Asif et al. 2020).

### One-step growth curve

The one-step growth curve was performed as previously described to determine the phage growth features (Cao et al. 2015). Briefly, 9 mL of host bacterium culture; *P. aeruginosa* PS28 was incubated with 90 µL of phage suspension at multiplicity of infection (MOI) of 0.1 (10^7^ PFU/mL) for 10 min to allow phage adsorption. Next, the mixture was centrifugated at 10,000 ×*g* for 10 min and the pellet was resuspended in 10 mL TS broth. Samples of 100 µL were collected at time intervals of 5 min over and subjected to phage titration by the double layer agar method. The assay was evaluated in triplicate and both phage latent period and burst size were determined. The phage burst size was determined as the ratio of the average number of free phage particles after the release phase to their number during the latency phase (Cao et al. 2015).

### In vitro killing assay

The lytic activity of isolated phage against both the host strain (PS28) and PAO1, a strain with high EOP ratio, was assayed. Phage suspensions at different MOIs (0.1, 1 and 10) were co-cultured with 10^8^ CFU/mL of bacterial suspensions and incubated with shaking at 37 °C. The inhibitory effect of isolated phage on bacterial growth was determined spectrophotometrically at OD_600_ and compared with bacterial culture without phage. Furthermore, the number of revival bacteria and phage were counted following phage infection (Morozova et al. 2022). Briefly, phage suspensions at different MOIs (0.1, 1 and 10) were co-cultured with bacterial suspensions and incubated overnight with shaking at 37 °C. Then, aliquots were taken, serially diluted and plated over TS agar plates. The assays were performed in triplicate and results were expressed as means ± standard errors (Chen et al. 2018).

### Biofilm inhibition assay

The ability of isolated phage to eradicate biofilms formed by *P. aeruginosa* was characterized (Liu et al. 2020). Bacterial cultures were allowed to form biofilm onto the surface of 96-well polystyrene U-shaped microtiter plate (Costar^™^; Corning^™^) exactly as described above. Following incubation, broth culture was gently decanted and wells were washed with sterile phosphate buffer saline (PBS) to remove the planktonic cells. Next, aliquotes of about 200 μL of phage suspension at various MOIs (0.1, 1 and 10) in TS broth were added to each well and incubated overnight at 37 °C. Control wells received sterile TS broth only without phage. The formed biofilms were assayed using the crystal violet assay and absorbance was measured spectrophotometrically at 570 nm. The experiment was performed in triplicate and results were expressed as means ± standard errors.

### Bacteriophage genome sequencing and data analysis

The phage vB_PaeP_PS28 nucleic acid was extracted from phage lysate (2.5 × 10^12^ PFU/mL) using QIAamp1 DNA Mini kit (QIAGEN, Germany) following the manufacturer guidelines and DNA pellet was stored at − 20 °C until use. The DNA library was prepared using the Nextera XT DNA Library preparation kit (Illumina, USA). The DNA was fragmented then tagged utilizing the transposome in the Nextera XT Kit. The whole genome sequencing was done by Illumina Miseq next-generation sequencing at Genomics and Epigenomics Program, Children’s Cancer Hospital Egypt, Cairo, Egypt. All preparations and the sequencing run were performed according to Illumina manufacturing instructions. The quality of paired-end DNA reads was evaluated using FASTQC (Brown et al. 2017). Moreover, low-quality bases were trimmed using Trimmomatic v0.36. The generated sequences were assembled using Unicycler v0.4.8 to assemble the reads into contigs (Wick et al. 2017). Assembly quality was checked using QUAST v 5.0.2 (Gurevich and Saveliev 2013). The number of open reading frames (ORFs) in phage genome was predicted and putative functions of predicted ORFs were annotated using Prokka v 1.14 (Seemann 2014). The circular genomic map of vB_PaeP_PS28 was generated and visualized via CGView (Stothard and Wishart 2005). The online tool tRNAscan-SE 1.21 was used to look for tRNA genes in the phage sequences (Chan and Lowe 2019). The phylogeny tree was generated using BLAST (Basic Local Alignment Search Tool) using the neighbor-joining method. Intergenomic similarity between isolated phage and other related *Pseudomonas* phages was calculated using VIRIDIC v1.1; the Virus Intergenomic Distance Calculator (Moraru et al. 2020). In addition, phylogenetic trees based on the terminase large subunit and RNA polymerase large subunit were constructed (Altschul et al. 1990). The Dot Plot analysis was performed using VectorBuilder’s Sequence Dot Plot tool and comparative analysis of the whole genome with other related phages was performed by Easyfig program (Sullivan et al. 2011).

### Assessment of the bacteriolytic activity of isolated phage in vivo using mice infection model

The influence of isolated phage on *P. aeruginosa* pathogenesis was characterized in vivo using mice infection model (Alvi et al. 2021). All procedures in animal infection experiment were performed according to ethical standards of the Zagazig University Institutional Animal and Use Committee (ZU-IACUC), which was granted Approval Number (ZU-IACUC/3/F/72/2022). Briefly, five groups (15 mice each; 6 mice for survival experiment and 9 mice for determining phage and bacterial load) of 4 weeks old albino mice were included in the experiment. The first group contained mice inoculated intraperitoneally (I.P) with *P. aeruginosa* at (2.5 × 10^7^ CFU/mL), the second group contained *Pseudomonas*-inoculated mice and treated intraperitoneally with isolated phage at MOI = 100 (2.5 × 10^9^ PFU/mL). The third group contained mice inoculated with isolated phage (2.5 × 10^9^ PFU/mL) only. In addition, both non-injected and PBS-injected mice groups were included as controls. Mice survival in each group was monitored daily over 4 days-period and plotted using Kaplan–Meier method using Log-rank test for statistical analysis. In addition to mice survival, three mice from each group were anesthetized and sacrificed at 24, 48 and 72 h post inoculation. Mice liver and spleen were aseptically obtained for determination of both bacterial burden and phage titer. Isolated organs were homogenized, serially diluted in PBS and plated on cetrimide agar plates for enumeration of bacterial CFUs. In addition, the homogenate was filtered, serially diluted and overlaid by the double layer agar method to determine the phage titer in treated mice and expressed as (PFUs). Both bacterial load and phage titer were determined and expressed as means ± standard errors. The statistical analysis was performed by Mann–Whitney U analysis with *P* < 0.05 was considered significant.

### Statistical analysis

Statistical analysis was performed using GraphPad Prism 5 software using Student t-tests or one-way ANOVA unless otherwise stated. All experiments were performed in triplicate and data expressed as the mean ± standard errors.

## Results

### Antibiotic susceptibility and assessment of biofilm formation by *P. aeruginosa* isolates

A total of 50 *P. aeruginosa* isolates obtained from different clinical sources; burns (10 isolates), surgical wounds (10 isolates), urine (11 isolates), ear infections (2 isolates) and endotracheal aspirates (17 isolates) were included in this study. The susceptibility profile of *P. aeruginosa* isolates against different antibiotics was determined by the disc diffusion method and results were interpreted according to CLSI (2018) guidelines **(**Additional file 1: Table S1). All *P. aeruginosa* isolates were sensitive to colistin while high bacterial resistance was observed towards gentamicin (60%). Majority of *P. aeruginosa* isolates exhibited high resistance to fluoroquinolone and carbapenems (58% and 54%; each). The antibiotic susceptibility results reveal that about of 56% of *P. aeruginosa* isolates were multi-drug resistant (MDR). In addition to antibiotic susceptibility, the biofilm forming capacity of *P. aeruginosa* isolates was assessed spectrophotometrically by the crystal violet assay. Bacterial isolates were categorized into 3 groups according to biofilm formation; strong (20.4% of bacterial isolates), moderate (64.8% of bacterial isolates) and weak biofilm forming (14.8% of bacterial isolates) as shown in (Additional file 1: Fig. S1).

### Phage isolation

**Fig. 1:**
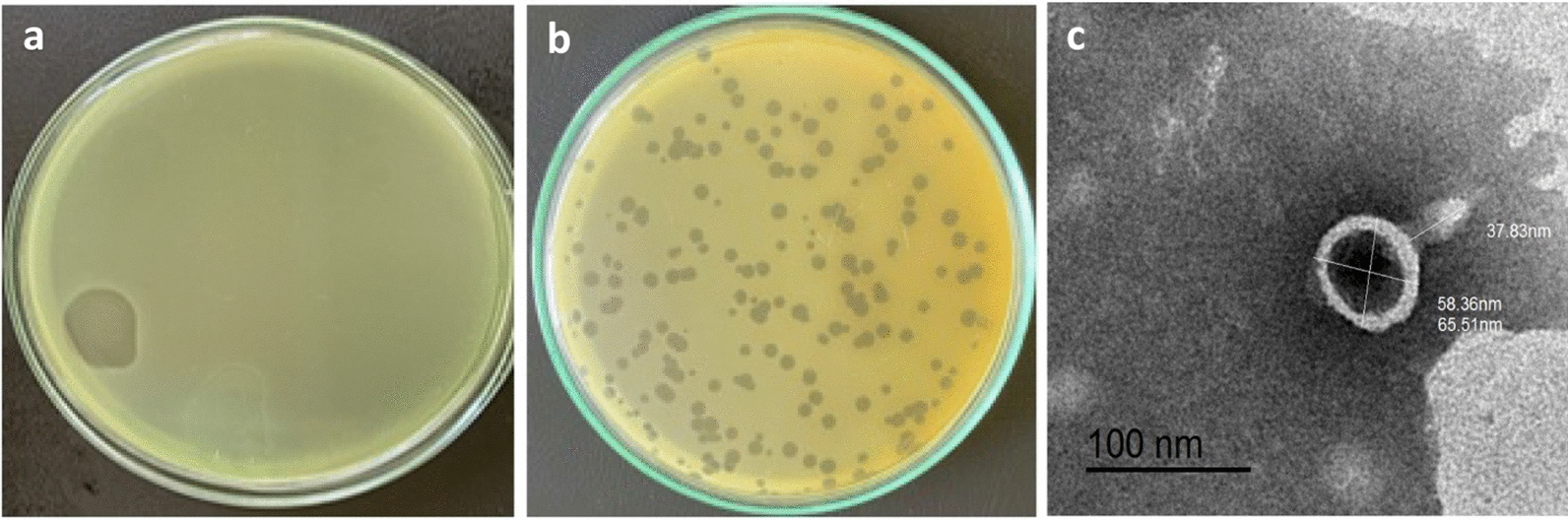
Isolation of bacteriophage. **a** Clear lytic zone on bacterial lawn by spot assay of phage lysate from sewage. **b** Plaque morphology of isolated phage double layer agar plate. **c** Transmission electron microscope (TEM) images of vB_PaeP_PS28. Phage particles were negatively stained by 2% phosphotungstic acid. Scale bar = 100 nm

A lytic phage specific for *P. aeruginosa* was isolated from sewage by the enrichment technique using *P. aeruginosa* PS28 as host strain. *P. aeruginosa* PS28 was found to be MDR strain and exhibit a strong biofilm-forming capability. The isolated phage was designated as vB_PaeP_PS28 according to the recommended nomenclature procedure (Kropinski et al. 2009). *P. aeruginosa* phage vB_PaeP_PS28 produced circular plaques with diameter of 2–3 mm in double layer agar method **(**Fig. 1a, b**)**. Plaques of homogenous morphology were selected, purified several rounds for further analysis and purified phage stock stored in SM buffer at 4 °C**.**

### Phage morphology characterization by transmission electron microscopy (TEM)

TEM images revealed that the phage vB_PaeP_PS28 possesses an icosahedral head and short non-contractile tail which are closely related to phages belonging to *Podoviridae* family according to International Committee Taxonomy of Viruses (ICTV) (Adriaenssens and Brister 2017). The phage head diameter is 65.5 nm and tail length of 37.8 nm that are extremely typical to the *Podoviridae* family within the order *Caudovirales* **(**Fig. 1c**)**.

### Host range and efficiency of plating (EOP) of the phage vB_PaeP_PS28

The bacteriolytic activity of vB_PaeP_PS28 against different *P. aeruginosa* strains and other bacterial species was evaluated by the spot assay. The phage vB_PaeP_PS28 has the ability to infect and lyse approximately 13/18 (72.2%) of tested *P. aeruginosa* strains which indicates a broad spectrum lytic activity of isolated phage **(**Table 1**)**. Moreover, the phage vB_PaeP_PS28 able to infect most of tested MDR *P. aeruginosa* strains (8/9) from different clinical sources such as burn, wound, urine, ear infections and endotracheal aspirates (Additional file 1: Table S2). However, no lytic activity was observed against other bacterial species by phage vB_PaeP_PS28. Furthermore, the susceptibility of tested isolates to the phage vB_PaeP_PS28 was confirmed by EOP analysis. Series of diluted phage vB_PaeP_PS28 were plated against susceptible strains. EOP values of phage-bacteria mixtures were varied into low (n = 3); medium (n = 4) and high EOP values (n = 6) **(**Table 1**)**.

**Table 1:** Host range and efficiency of plating (EOP) of phage vB_PaeP_PS28

Bacterial isolate^b^	vB_PaeP_PS28		
	Infectivity^a^	EOP ratio (mean ± SD)	Interpretation
PS 3B	+	0.03 ± 0.003	Low
PS 6B	+	0.23 ± 0.05	Medium
PS 9B	−	–	–
PS 10B	+	0.35 ± 0.05	Medium
PS 11W	−	–	–
PS 13W	+	0.62 ± 0.04	High
PS 14W	+	1.15 ± 0.1	High
PS 22U	+	0.34 ± 0.04	Medium
PS 23U	+	0.06 ± 0.005	Low
PS 24U	+	0.43 ± 0.04	Medium
PS 28U	+	1	High (host)
PS 32SP	+	0.8 ± 0.02	High
PS 38SP	+	0.04 ± 0.004	Low
PS 41SP	−	–	–
PS 49E	−	–	–
*P. aeruginosa* PAO1	+	1.25 ± 0.05	High
*P. aeruginosa* ATCC 27853	+	0.7 ± 0.03	High
*P. aeruginosa* ATCC 9027	−	–	–
*E. coli* ATCC 10536	−	–	–
*E. coli* ATCC O26	−	–	–
*E. coli* ATCC O78	−	–	–
*E. coli* ATCC O157	−	–	–
*S.* Typhimurium ATCC 14028	−	–	–
*K. pneumoniae* ATCC 700603	−	–	–
*Serratia marcescens*	−	–	–
*S. aureus* ATCC 6538	−	–	–
*S. aureus* ATCC 9295	−	–	–

^**a**^ + indicates presence of clear zone (lysis) and -: indicates no lysis was observed

^**b**^*B* burn, *W* surgical wound, *U* urine, *SP* endotracheal aspirates

### Temperature and pH stability of the phage vB_PaeP_PS28

**Fig. 2:**
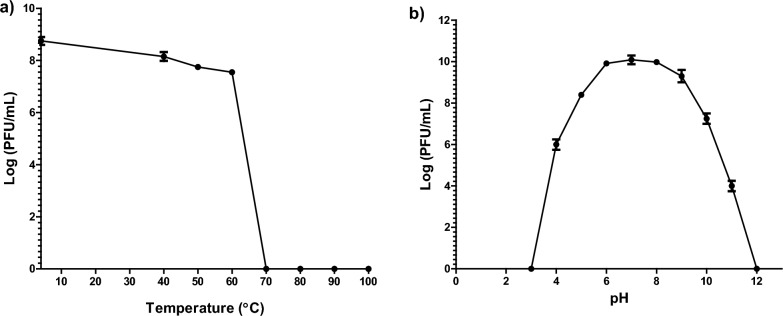
Physical properties of vB_PaeP_PS28. **a** Thermal stability; **b** pH stability. Error bars represent mean ± SE for three replicates

The thermal stability of vB_PaeP_PS28 was assessed by monitoring change in phage titer upon incubation under different temperatures. The results indicate that the phage vB_PaeP_PS28 is tolerant to wide range of temperatures. Isolated phage could survive up to 60 °C with no significant reduction in phage titer. However, there was a significant reduction in phage titer upon incubation of vB_PaeP_PS28 at higher temperatures (70, 80, 90 and 100 °C) as shown in Fig. 2a. Regarding phage pH stability, the phage vB_PaeP_PS28 was able to retain its infectivity and could survive at pH ranges from 4 to 10. There was a slight reduction in phage viability at pH 11 and viable phage particles were not observed at extreme pH (3 and 12) **(**Fig. 2b**)**.

### One-step growth curve and in vitro killing assay

**Fig. 3:**
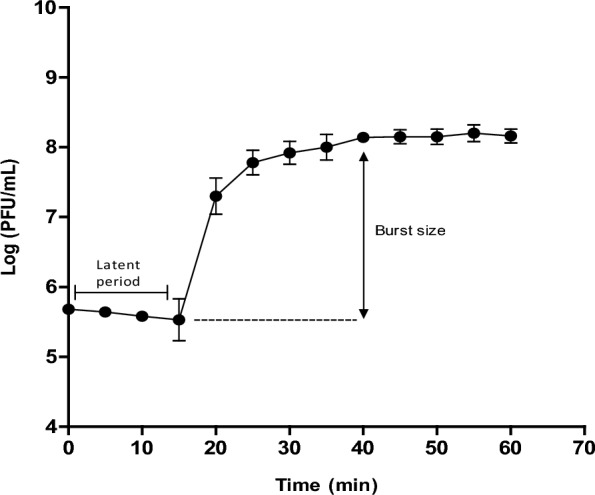
One-step growth curve of vB_PaeP_PS28. Phage was incubated with exponential culture of PS28 for 10 min, centrifuged and pellet was resuspended in TS broth. Titer of free phages was determined by double layer agar technique. Three biological replicates were performed and data were presented as mean ± SE

**Fig. 4:**
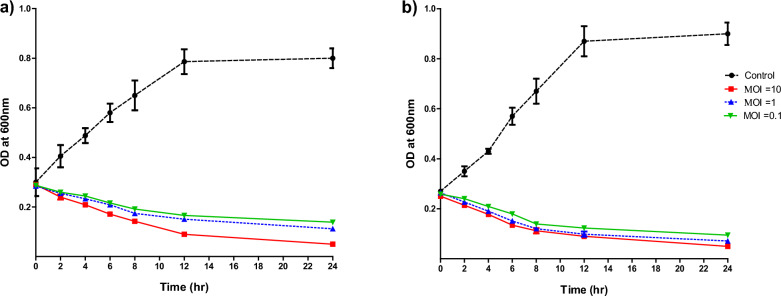
Bacteriolytic activity of phage vB_PaeP_PS28 against the host strain *P. aeruginosa* PS28 (**a**) and PAO1 **b**. Early exponential bacterial cultures were incubated with and without isolated phage suspension at MOI of (0.1, 1 and 10) at 37 °C for 24 h. Bacterial growth was determined and measured spectrophotometrically at OD_600_. The results were expressed as means ± SE of three independent experiments

The one-step growth curve revealed that the phage vB_PaeP_PS28 has a latent period of 15 min and an average burst size of 210 virions per infected bacterium **(**Fig. 3**)**. The bacteriolytic activity of vB_PaeP_PS28 at different MOIs (0.1, 1 and 10) was determined against both the phage host strain PS28 and PAO1. As shown in Fig. 4, control culture without phage treatment continued to grow during the incubation period. On the other hand, the phage vB_PaeP_PS28 was able to adversely affect bacterial growth over 24 h. Importantly, bacterial growth inhibition was found to be dose dependent where growth inhibition was higher at MOI of 10 as compared to MOI of 1 and 0.1. In addition, the vB_PaeP_PS28 phage could effectively reduce the number of surviving bacterial count in MOI dependent manner (Additional file 1: Table S3). Thus, the obtained data demonstrated high lytic activity of isolated phage.

### Biofilm inhibition assay

**Fig. 5:**
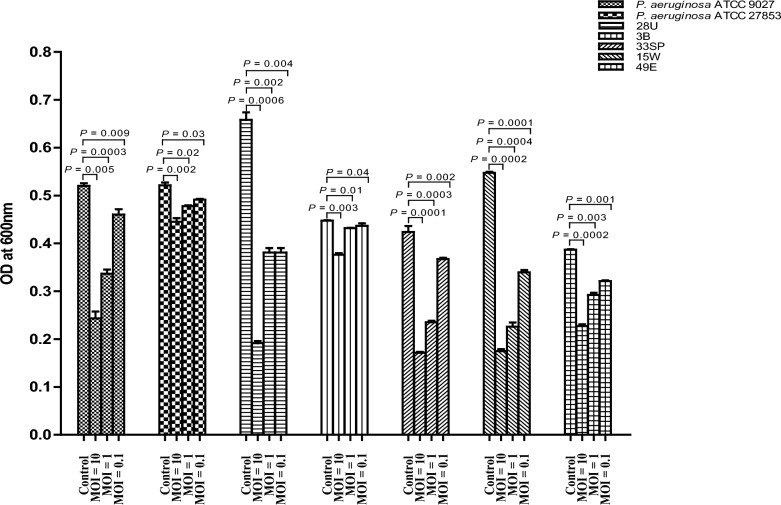
The antibiofilm activity of vB_PaeP_PS28 against various *P. aeruginosa* isolates. Biofilms were formed in 96-well plates for 24 h and treated with phage at different MOIs (0.1, 1 and 10) for 24 h. Formed biofilms were stained by 1% crystal violet and measured spectrophotometrically at OD_600_. The experiment was carried out at three independent replicates and data was expressed as means ± SE with *P* < 0.05 was considered significant

The antibiofilm activity of vB_PaeP_PS28 against five strong biofilm forming clinical isolates including its host strain PS28 as well as *P. aeruginosa* ATCC 9027 and ATCC 27853 was assessed by the crystal violet assay. The phage vB_PaeP_PS28 effectively degraded mature biofilms and reduced biofilm biomass formed by all tested *P. aeruginosa* strains. As shown in Fig. 5, the antibiofilm activity of vB_PaeP_PS28 against *P. aeruginosa* was MOI dependent where maximum biofilm inhibition was observed at MOI of 10 as compared with MOI of 1 and 0.1.

### Phage genome features

**Fig. 6:**
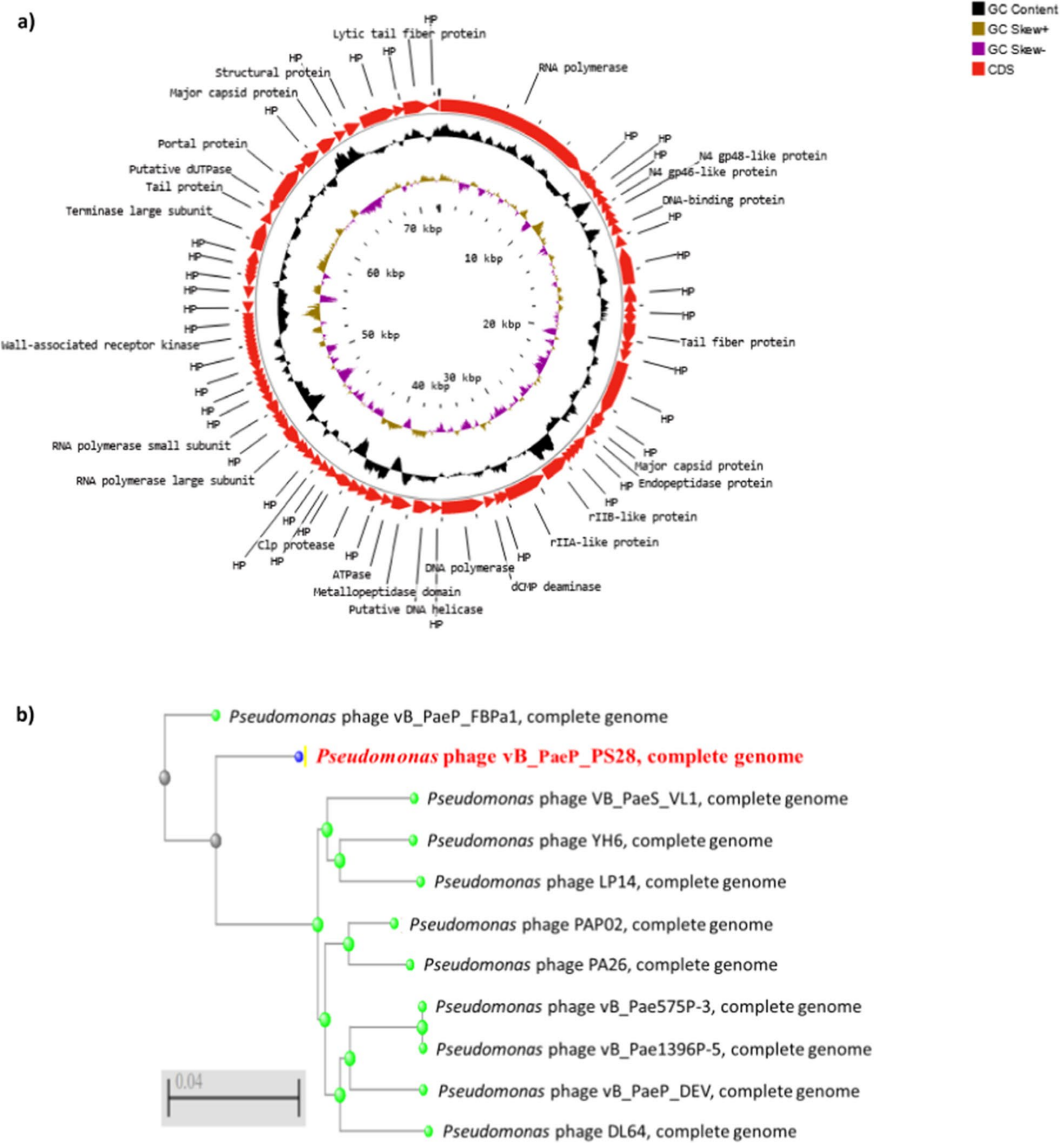

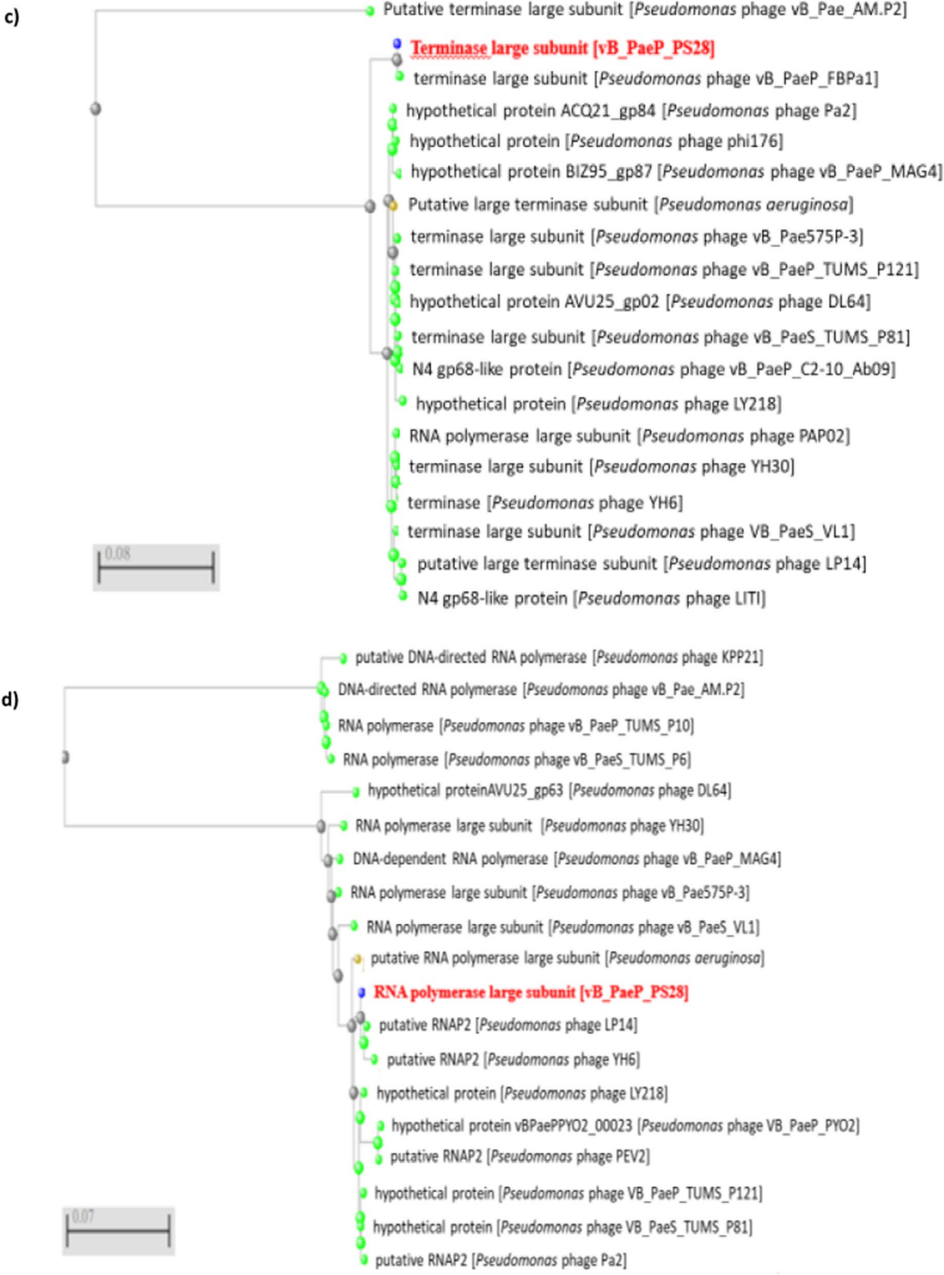
Genomic characterization of vB_PaeP_PS28. **a** Circular genomic map of vB_PaeP_PS28; from inside to outside, the first to third circles represent the scale, GC Skew, and GC content respectively; the fourth represents the position of ORFs. The prediction and direction of ORFs are indicated by arrow heads. The genomic map was generated and visualized using CGView **b** Phylogenetic analysis of vB_PaeP_PS28 and other closely related sequences. **c** Phylogenetic tree analysis based on the amino acid sequence of terminase large subunit. **d** Phylogenetic tree analysis based on the amino acid sequence of RNA polymerase large subunit. Phylogenetic trees were constructed using BLAST (Basic Local Alignment Search Tool) using neighbor-joining method

**Fig. 7:**
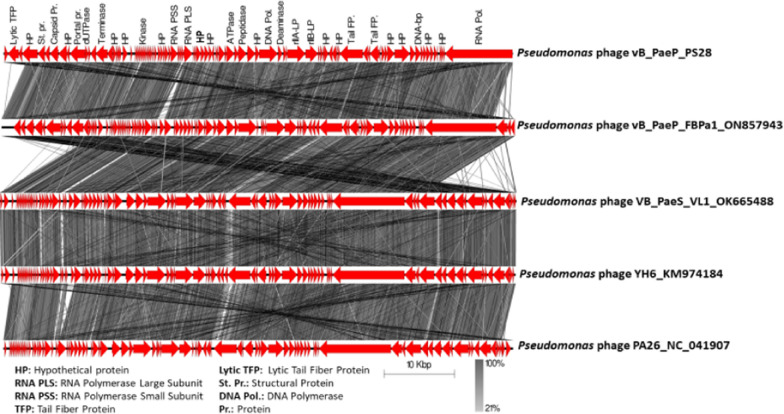
Comparative genomic analysis between phage vB_PaeP_PS28 and related sequences. *Pseudomonas* phage vB_PaeP_FBPa1 (GenBank Acc. No. ON857943.1), *Pseudomonas* phage VB_PaeS_VL1 (GenBank Acc. No. OK665488.1), *Pseudomonas* phage YH6 (GenBank Acc. No. KM974184.1) and *Pseudomonas* phage PA26 (GenBank Acc. No. NC_041907.1). Sequence similarity is represented by the gray scale bar. The coding sequences are represented by directional arrows. Predicted ORFs in vB_PaeP_PS28 genome are listed below. Comparative analysis was performed using Easyfig

**Fig. 8:**
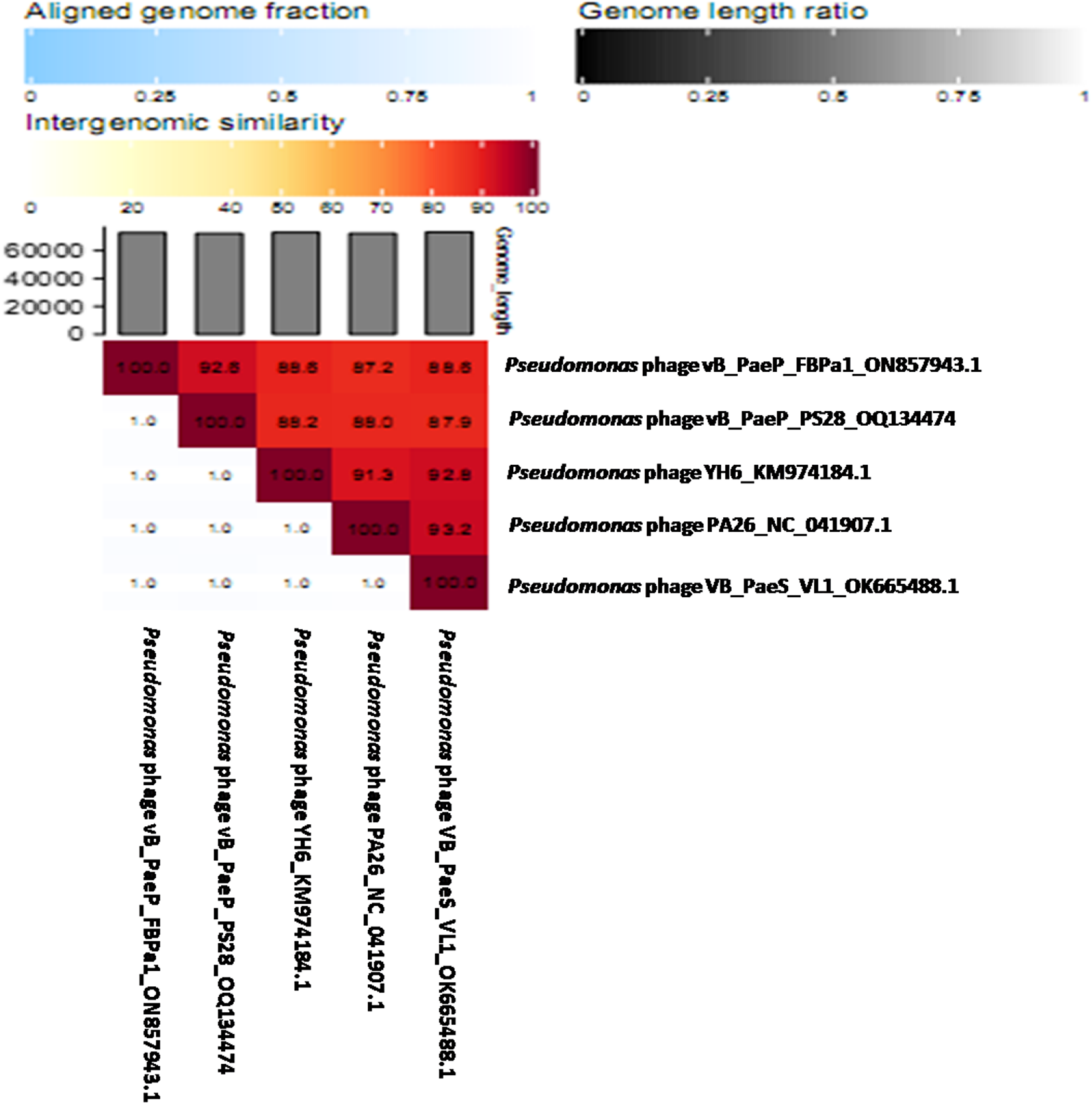
VIRIDIC heatmap of vB_PaeP_PS28 phage and its closest homologues. Intergenomic similarities between the genomic nucleotide sequences of vB_PaeP_PS28 and related bacteriophages infecting *P. aeruginosa*; *Pseudomonas* phage vB_PaeP_FBPa1 (GenBank Acc. No. ON857943.1), *Pseudomonas* phage YH6 (GenBank Acc. No. KM974184.1), *Pseudomonas* phage PA26 (GenBank Acc. No. NC_041907.1) and *Pseudomonas* phage VB_PaeS_VL1 (GenBank Acc. No. OK665488.1). Color coding scales are represented above the matrix with intensity of color corresponding to level of similarity

The whole genome of vB_PaeP_PS28 was sequenced by Illumina Miseq and assembly was performed by Unicycler v0.4.8. The phage vB_PaeP_PS28 genome is composed of 72,283 bp of circular double-stranded DNA, with 54.75% G + C content. The entire genome structure of vB_PaeP_PS28 is shown in Fig. 6a. In total, 94 ORFs were found, including only 32 ORFs (34%) were predicted to encode for functional proteins, whereas 62 ORFs (66%) were annotated as hypothetical proteins. The predicted functions of 32 ORFs were divided into 5 major modules including structure proteins (ORF 8, ORF 9, ORF 18, ORF 21, ORF 24, ORF 88, ORF 90 and ORF 93), DNA metabolism and replication (ORF 1, ORF 11, ORF 34, ORF 35, ORF 37, ORF 52, ORF 56, ORF 59, ORF 69, ORF 71 and ORF 84), packaging and assembly proteins (ORF 27, ORF 32, ORF 81, ORF 82, ORF 83 and ORF 85), host cell lysis modules (ORF 25, ORF 38, ORF 42 and ORF 51) and additional functions (ORF 30, ORF 31 and ORF 40) **(**Table 2**)**. ORFs 30 and 31 play a role in cell lysis inhibition and interference with cell metabolism. This would delay releasing of phage holin enzyme until the phage particles are formed and accumulated within the host. On the other hand, ORF 40 is involved in energy-requiring activities such as phage DNA packaging replication. The phage genome did not reveal any lysogenic genes or host genome-related sequences, so that vB_PaeP_PS28 referred to be a lytic phage. The genes related to antibiotic resistance in *P. aeruginosa*, host virulence factors and toxin genes were also absent in vB_PaeP_PS28 phage genome. The phylogenetic analysis **(**Fig. 6b**)** shows that vB_PaeP_PS28 is closely related to *Pseudomonas* phage vB_PaeP_FBPa1 (GenBank Acc. No ON857943.1), which is a member of the family *Podoviridae* and the genus *Litunavirus*. Additionally, phylogenetic trees were created for some of the predicted essential phage proteins; the terminase large subunit **(**Fig. 6c**)** and RNA polymerase large subunit **(**Fig. 6d**)**. Importantly, the genomic sequence similarity of the phage vB_PaeP_PS28 to other previously characterized phages infecting *P. aeruginosa* was determined. As shown in (Table 3, Fig. 7 and Additional file 1: Fig. S2), greatest similarity was observed between the phage vB_PaeP_PS28 and *Pseudomonas* phage vB_PaeP_FBPa1 (GenBank Acc. No. ON857943.1, identity, 94.81%); *Pseudomonas* phage VB_PaeS_VL1 (GenBank Acc. No. OK665488.1, identity, 94.37%); *Pseudomonas* phage YH6 (GenBank Acc. No. KM974184.1, identity, 94.07%); and *Pseudomonas* phage PA26 (GenBank Acc. No. NC_041907.1, identity, 94.04%). To further examine the taxonomy of the vB_PaeP_PS28 phage, matrix of intergenomic similarities of the vB_PaeP_PS28 genome with four most similar phage genomes *Pseudomonas* phage vB_PaeP_FBPa1, *Pseudomonas* phage VB_PaeS_VL1, *Pseudomonas* phage YH6 and *Pseudomonas* phage PA26 was calculated using VIRIDIC **(**Fig. 8). The genome sequence of the phage vB_PaeP_PS28 has been deposited in the GenBank database under GenBank Acc. No. OQ134474.

**Table 2:** Predicted ORFs identified in phage vB_PaeP_PS28 phage

Coding sequence	Start…..End	GC (%)	Protein length	MW (KDa)	Gene name	Putative function	Amino acid sequence identity/similarity to best homologs	BLAST score (E-Value)	Accession No.
ORF1	51…9608	56.25	3185	347.48	RNA polymerase	Transcription process and DNA replication	Virion-associated RNA polymerase [Pseudomonas phage PAP02]	0	QKE55114.1
ORF2	9624....9827	50.00	67	7.42	Hypothetical protein	Unknown function	Hypothetical protein FG40_gp65 [Pseudomonas phage vB_PaeP_C2-10_Ab09]	2e-07	YP_009031842.1
ORF3	9837…10154	57.55	105	11.47	Hypothetical protein	Unknown function	Hypothetical protein PP-LIT1_gp69 [Pseudomonas phage LIT1]	3e-70	YP_003358466.1
ORF4	10,15….10,387	55.27	78	8.88	Hypothetical protein	Unknown function	Hypothetical protein PAP02_040 [Pseudomonas phage PAP02]	1e-48	QKE55111.1
ORF5	10,687…10842	47.44	51	5.76	Hypothetical protein	Unknown function	Hypothetical protein [Pseudomonas phage LP14]	3e-27	AWY02728.1
ORF6	10,793….11197	57.04	134	14.52	Hypothetical protein	Unknown function	Hypothetical protein ACQ34_gp70 [Pseudomonas phage YH6]	7e-94	YP_009152570.1
ORF7	11,255…11692	62.79	145	14.64	Hypothetical protein	Unknown function	Hypothetical protein vB_Pae575P-3_65 [Pseudomonas phage vB_Pae575P-3]	1e-39	ANT44344.1
ORF8	11,704…12264	54.19	186	20.41	N4 gp46-like protein	Structural protein (tubular tail A homologue)	N4 gp46-like protein [Pseudomonas phage LIT1]	1e-126	YP_003358462.1
ORF9	12,252….12695	56.31	147	17.15	N4 gp48-like protein	Structural protein (major capsid)	N4 gp48-like protein [Pseudomonas phage LIT1]	1e-87	YP_003358461.1
ORF10	12,692…13057	55.19	121	13.83	Hypothetical protein	Unknown function	Hypothetical protein BIZ95_gp65 [Pseudomonas phage vB_PaeP_MAG4]	2e-83	YP_009290599.1
ORF11	13,061…13807	58.90	248	26.78	Putative single-stranded DNA-binding protein	DNA replication	Putative single-stranded DNA-binding protein [Pseudomonas aeruginosa]	1e-157	SBT96838.2
ORF12	13,834…14568	58.90	244	28.08	Hypothetical protein	Unknown function	Hypothetical protein FDH24_gp59 [Pseudomonas phage PA26]	0.0	YP_009598413.1
ORF13	14,617…16785	58.90	722	82.15	Hypothetical protein	Unknown function	Hypothetical protein BIZ95_gp62 [Pseudomonas phage vB_PaeP_MAG4]	0.0	YP_009290596.1
ORF14	16,800…17810	54.60	336	37.8	Hypothetical protein	Unknown function	Hypothetical protein FBPa1_0077 [Pseudomonas phage vB_PaeP_FBPa1]	0.0	UVN14432.1
ORF15	17,812…18192	56.69	126	14.4	Hypothetical protein	Unknown function	Hypothetical protein [Pseudomonas phage vB_PaeS_TUMS_P81]	6e-81	UGL60992.1
ORF16	18,189…18617	57.58	142	15.76	Hypothetical protein	Unknown function	Hypothetical protein ACQ34_gp60 [Pseudomonas phage YH6]	4e-99	YP_009152560.1
ORF17	18,820…18990	53.80	56	6.38	Hypothetical protein	Unknown function	Hypothetical protein FBPa1_0074 [Pseudomonas phage vB_PaeP_FBPa1]	1e-30	UVN14429.1
ORF18	19,011…20300	52.44	429	46.32	Tail fiber protein	Phage assembly (Tail morphogenesis)	Tail fiber protein [Pseudomonas phage vB_Pae575P-3]	0.0	ANT44334.1
ORF19	20,297…20596	50.67	99	11.7	Hypothetical protein	Unknown function	Hypothetical protein FBPa1_0072 [Pseudomonas phage vB_PaeP_FBPa1]	4e-65	UVN14427.1
ORF20	20,593…21264	50.45	223	24.96	Hypothetical protein	Unknown function	Hypothetical protein FBPa1_0071 [Pseudomonas phage vB_PaeP_FBPa1]	4e-161	UVN14426.1
ORF21	21,303…24560	55.03	1085	118.09	Tail fiber protein	Phage assembly (Tail morphogenesis)	Putative tail fiber protein [Pseudomonas phage PEV2]	0.0	YP_009286275.1
ORF22	24,488…24781	49.66	97	11.17	Hypothetical protein	Unknown function	Hypothetical protein BIZ95_gp54 [Pseudomonas phage vB_PaeP_MAG4]	1e-50	YP_009290588.1
ORF23	24,915…25109	55.38	64	7.52	Hypothetical protein	Unknown function	Hypothetical protein BIZ95_gp53 [Pseudomonas phage vB_PaeP_MAG4]	4e-33	YP_009290587.1
ORF24	25,106…25630	58.29	174	19.6	Major capsid protein	Phage structural assembly (Head morphogenesis)	Major capsid protein [Pseudomonas phage vB_PaeP_FBPa1]	1e-123	UVN14423.1
ORF25	25,627…26139	56.14	170	18.68	Endopeptidase protein	Host cell lysis gene	Endopeptidase protein [Pseudomonas phage PAP02]	1e-103	QKE55097.1
ORF26	26,202…26759	52.51	185	21.36	Hypothetical protein	Unknown function	Hypothetical protein vBPaeSVL1_52 [Pseudomonas phage VB_PaeS_VL1]	1e-128	UGV19848.1
ORF27	26,752….26943	50.00	63	7.2	HNH endonuclease	Phage DNA packaging	HNH endonuclease [Pseudomonas phage YH30]	1e-32	YP_009226156.1
ORF28	26,993…27310	51.57	105	11.84	Hypothetical protein	Unknown function	Hypothetical protein PP-LIT1_gp45 [Pseudomonas phage LIT1]	3e-69	YP_003358442.1
ORF29	27,394…27618	50.67	74	8.86	Hypothetical protein	Unknown function	Hypothetical protein FBPa1_0063 [Pseudomonas phage vB_PaeP_FBPa1]	2e-35	UVN14418.1
ORF30	27,666….29435	60.17	589	63.4	Putative rIIB-like protein	Lysis inhibition, interfere with cell metabolism	Putative rIIB-like protein [Pseudomonas phage VB_PaeS_VL1]	0.0	UGV19844.1
ORF31	29,447…31975	55.36	842	95.11	Putative rIIA-like protein	Lysis inhibition, interfere with cell metabolism	Putative rIIA-like protein [Pseudomonas phage YH6]	0.0	YP_009152547.1
ORF32	31,979…32170	56.77	63	7.2	HNH endonuclease	Phage DNA packaging	HNH endonuclease [Pseudomonas phage YH6]	5e-40	YP_009152546.1
ORF33	32,167…32565	55.39	132	14.55	Hypothetical protein	Unknown function	Hypothetical protein FBPa1_0059 [Pseudomonas phage vB_PaeP_FBPa1]	5e-78	UVN14414.1
ORF34	32,740…33309	55.61	189	20.62	Putative dCMP deaminase	Nucleotide metabolism and DNA replication	Putative dCMP deaminase [Pseudomonas phage VB_PaeS_VL1]	2e-113	UGV19839.1
ORF35	33,391…36006	54.51	871	97.96	Putative DNA polymerase	DNA replication	Putative DNA polymerase [Pseudomonas phage LP14]	0.0	AWY02758.1
ORF36	36,006…36533	54.92	175	19.98	Hypothetical protein	Unknown function	Hypothetical protein PAP02_016 [Pseudomonas phage PAP02]	2e-125	QKE55087.1
ORF37	36,533…37699	56.64	388	44.08	Putative DNA helicase	DNA replication	Putative DNA helicase [Pseudomonas phage Pa2]	0.0	YP_009148216.2
ORF38	37,789…39015	56.40	408	45.58	Putative metallopeptidase domain	Lysis bacterial cell wall peptidoglycan	Putative metallopeptidase domain [Pseudomonas phage YH6]	0.0	YP_009152539.1
ORF39	39,015…39527	46.59	170	18.9	Hypothetical protein	Unknown function	Hypothetical protein FBPa1_0052 [Pseudomonas phage vB_PaeP_FBPa1]	7e-121	UVN14407.1
ORF40	39,539…40609	57.80	356	40.04	ATPase	Energy production during phage DNA packaging	ATPase [Pseudomonas phage YH30]	0.0	YP_009226170.1
ORF41	40,641…40883	60.08	80	9.02	Hypothetical protein	Unknown function	Hypothetical protein FBPa1_0050 [Pseudomonas phage vB_PaeP_FBPa1]	6e-52	UVN14405.1
ORF42	40,880…41713	57.67	277	30.99	ATP-dependent Clp protease ATP-binding subunit	Energy dependent protein degradation	ATP-dependent Clp protease ATP-binding subunit [Pseudomonas phage VB_PaeS_VL1]	0.0	UGV19832.1
ORF43	41,772…41957	49.46	61	7.07	Hypothetical protein	Unknown function	Hypothetical protein FDH24_gp30 [Pseudomonas phage PA26]	4e-37	YP_009598384.1
ORF44	41,966…42196	44.16	76	8.4	Hypothetical protein	Unknown function	Hypothetical protein vB_pae575P-3_30a [Pseudomonas phage vB_Pae575P-3]	2e-31	ANT44308.1
ORF45	42,434…42730	55.56	98	11.44	Hypothetical protein	Unknown function	Hypothetical protein BIZ95_gp30 [Pseudomonas phage vB_PaeP_MAG4]	5e-45	YP_009290564.1
ORF46	42,652…43161	54.31	169	18.96	Hypothetical protein	Unknown function	Hypothetical protein [Pseudomonas phage LY218]	3e-65	QHZ59466.1
ORF47	43,158…43739	53.44	193	21.97	Hypothetical protein	Unknown function	Hypothetical protein [Pseudomonas phage LP14]	1e-90	AWY02768.1
ORF48	43,739…44293	54.41	184	21.71	Hypothetical protein	Unknown function	Hypothetical protein FDH24_gp26 [Pseudomonas phage PA26]	3e-129	YP_009598380.1
ORF49	44,290…44916	52.95	208	24.47	Hypothetical protein	Unknown function	Hypothetical protein BIZ95_gp26 [Pseudomonas phage vB_PaeP_MAG4]	3e-142	YP_009290560.1
ORF50	44,920…45144	58.67	74	8.18	Hypothetical protein	Unknown function	Hypothetical protein FBPa1_0041 [Pseudomonas phage vB_PaeP_FBPa1]	8e-19	UVN14396.1
ORF51	45,217…45369	49.67	50	5.7	Membrane protein	Lysis protein	Membrane protein [Pseudomonas phage PAP02]	2e-27	QKE55073.1
ORF52	45,467…46708	55.72	413	47.13	RNA polymerase large subunit	Transcription and mRNA processing	Putative RNA polymerase II (RNAP2) [Pseudomonas phage Pa2]	0.0	ANT44300.1
ORF53	46,740…47015	42.39	91	10.73	Hypothetical protein	Unknown function	Hypothetical protein PAP02_080 [Pseudomonas phage PAP02]	3e-09	QKE55151.1
ORF54	47,020…47310	43.99	96	11.48	Hypothetical protein	Unknown function	Hypothetical protein FBPa1_0037 [Pseudomonas phage vB_PaeP_FBPa1]	1e-54	UVN14392.1
ORF55	47,315…47575	49.81	86	10.08	Hypothetical protein	Unknown function	Hypothetical protein vB_Pae575P-3_21 [Pseudomonas phage vB_Pae575P-3]	7e-53	ANT44297.1
ORF56	47,588…48520	50.16	310	35.94	RNA polymerase small subunit	Transcription of phage protein	RNA polymerase small subunit [Pseudomonas phage YH30]	0.0	YP_009226181.1
ORF57	48,532…48882	48.72	116	13.49	Hypothetical protein	Unknown function	Hypothetical protein FBPa1_0034 [Pseudomonas phage vB_PaeP_FBPa1]	7e-79	UVN14389.1
ORF58	48,915…49355	56.92	146	16.18	Hypothetical protein	Unknown function	Hypothetical protein FBPa1_0033 [Pseudomonas phage vB_PaeP_FBPa1]	2e-105	UVN14388.1
ORF59	49,355…49606	54.37	83	9.62	Transcriptional regulator	Transcriptional regulator	Transcriptional regulator [Pseudomonas phage YH30]	6e-50	YP_009226097.1
ORF60	49,606…50007	57.46	133	14.48	Hypothetical protein	Unknown function	Hypothetical protein [Pseudomonas phage LP14]	1e-82	AWY02783.1
ORF61	50,007…50372	54.64	121	13.24	Hypothetical protein	Unknown function	Hypothetical protein FBPa1_0030 [Pseudomonas phage vB_PaeP_FBPa1]	2e-56	UVN14385.1
ORF62	50,557…50847	61.86	96	10.65	Hypothetical protein	Unknown function	Hypothetical protein FG40_gp12 [Pseudomonas phage vB_PaeP_C2-10_Ab09]	2e-60	YP_009031789.1
ORF63	50,939…51124	56.99	61	6.91	Hypothetical protein	Unknown function	Hypothetical protein FBPa1_0028 [Pseudomonas phage vB_PaeP_FBPa1]	1e-35	UVN14383.1
ORF64	51,197…51418	53.60	73	8.03	Hypothetical protein	Unknown function	Hypothetical protein [Pseudomonas phage CMS1]	5e-41	UNY40717.1
ORF65	51,415…51648	56.84	77	8.57	Hypothetical protein	Unknown function	Hypothetical protein [Pseudomonas phage phi176]	3e-44	AIZ94943.1
ORF66	51,645…51866	59.01	73	7.97	Hypothetical protein	Unknown function	Hypothetical protein PAP02_067 [Pseudomonas phage PAP02]	6e-31	QKE55138.1
ORF67	51,895…52098	56.37	67	7.3	Hypothetical protein	Unknown function	Hypothetical protein FBPa1_0024 [Pseudomonas phage vB_PaeP_FBPa1]	2e-28	UVN14379.1
ORF68	52,102…52398	54.21	98	11.26	Hypothetical protein	Unknown function	Hypothetical protein FBPa1_0023 [Pseudomonas phage vB_PaeP_FBPa1]	3e-63	UVN14378.1
ORF69	52,382…52690	50.81	102	12.08	Wall-associated receptor kinase-like 20-like protein	DNA metabolism and replication	Wall-associated receptor kinase-like 20-like protein [Pseudomonas phage YH30]	1e-65	YP_009226107.1
ORF70	52,687…52938	47.62	83	9.83	Hypothetical protein	Unknown function	Hypothetical protein P3P1_05 [Pseudomonas aeruginosa]	3e-35	SBT96754.2
ORF71	52,935…53168	49.15	77	8.95	Putative transcriptional regulator	Transcriptional regulator	Putative transcriptional regulator [Pseudomonas phage VB_PaeS_VL1]	2e-28	UGV19802.1
ORF72	53,165…53395	50.22	76	8.71	Hypothetical protein	Unknown function	Hypothetical protein FBPa1_0021 [Pseudomonas phage vB_PaeP_FBPa1]	3e-49	UVN14376.1
ORF73	53,410…53601	48.96	63	7.3	Hypothetical protein	Unknown function	Hypothetical protein FBPa1_0020 [Pseudomonas phage vB_PaeP_FBPa1]	2e-39	UVN14375.1
ORF74	54,016…54270	52.55	84	9.6	Hypothetical protein	Unknown function	Hypothetical protein FG40_gp01 [Pseudomonas phage vB_PaeP_C2-10_Ab09]	1e-50	YP_009031778.1
ORF75	54,691…55341	48.39	216	24.19	Hypothetical protein	Unknown function	Hypothetical protein [Pseudomonas phage LP14]	5e-155	AWY02705.1
ORF76	55,543…55851	57.93	102	11.45	Hypothetical protein	Unknown function	Hypothetical protein FDH24_gp87 [Pseudomonas phage PA26]	3e-57	YP_009598441.1
ORF77	55,827….56102	53.62	91	10.68	Hypothetical protein	Unknown function	Hypothetical protein [Pseudomonas phage vB_PaeP_TUMS_P121]	4e-34	UEP18636.1
ORF78	56,099….56311	50.23	70	7.73	Hypothetical protein	Unknown function	Hypothetical protein FBPa1_0014 [Pseudomonas phage vB_PaeP_FBPa1]	5e-38	UVN14369.1
ORF79	56,311….56742	53.70	143	16.1	Hypothetical protein	Unknown function	Hypothetical protein BIZ95_gp89 [Pseudomonas phage vB_PaeP_MAG4]	2e-93	YP_009290623.1
ORF80	56,809…57537	53.36	242	27.32	Hypothetical protein	Unknown function	Hypothetical protein FBPa1_0012 [Pseudomonas phage vB_PaeP_FBPa1]	2e-179	UVN14367.1
ORF81	57,534….59186	53.18	550	62.77	Terminase large subunit	Packing process and phage assembly	Terminase large subunit [Pseudomonas phage vB_PaeP_FBPa1]	0.0	UVN14366.1
ORF82	59,183…59917	50.88	244	27.91	Putative tail protein	Phage assembly (Tail morphogenesis)	Putative tail protein [Pseudomonas phage YH30]	8e-178	YP_009226120.1
ORF83	59,950….60249	56.00	99	11.1	ABC transporter-like protein	Mediate translocation to cell surface	ABC transporter-like protein [Pseudomonas phage YH30]	5e-63	YP_009226121.1
ORF84	60,253…60672	55.00	139	14.98	Putative dUTPase	DNA replication and metabolism	Putative dUTPase [Pseudomonas phage PEV2]	8e-94	YP_009286305.1
ORF85	60,707…62887	54.20	726	81.66	Portal protein	Translocation of phage DNA and packaging process (virion assembly)	Portal protein [Pseudomonas phage DL64]	0.0	YP_009206215.1
ORF86	62,957….63295	56.05	112	13.08	Hypothetical protein	Unknown function	Hypothetical protein PP-LIT1_gp79 [Pseudomonas phage LIT1]	4e-65	YP_003358476.1
ORF87	63,295….64476	52.28	393	44.13	Hypothetical protein	Unknown function	Hypothetical protein vBPaeSVL1_82 [Pseudomonas phage VB_PaeS_VL1]	0.0	UGV19878.1
ORF88	64,511…65710	59.75	399	44.06	Major capsid protein	Phage structural assembly (Head morphogenesis)	Major capsid protein [Pseudomonas phage VB_PaeS_VL1]	0.0	UGV19877.1
ORF89	65,767….66432	59.01	221	25.02	Hypothetical protein	Unknown function	Hypothetical protein vB_Pae575P-3_75 [Pseudomonas phage vB_Pae575P-3]	1e-123	ANT44354.1
ORF90	66,436…67401	55.49	321	35.21	Structural protein	Structural protein	Structural protein [Pseudomonas phage vB_Pae575P-3]	0.0	ANT44353.1
ORF91	67,459…69681	53.13	740	82.22	Hypothetical protein	Unknown function	Hypothetical protein [Pseudomonas phage vB_PaeS_TUMS_P81]	0.0	UGL60976.1
ORF92	69,662…70129	57.26	155	16.64	Hypothetical protein	Unknown function	Hypothetical protein FBPa1_0091 [Pseudomonas phage vB_PaeP_FBPa1]	3e-95	UVN14446.1
ORF93	70,129…71694	55.43	521	57.27	Putative lytic tail fiber protein	Structural protein (Tail fiber morphogenesis)	Lytic tail fiber [Pseudomonas phage YH30]	0.0	YP_009226131.1
ORF94	71,674…72150	57.02	158	16.78	Hypothetical protein	Unknown function	Hypothetical protein [Pseudomonas phage LY218]	4e-65	QHZ59508.1

**Table 3:** Homology of vB_PaeP_PS28 phage to other phages genomes

Scientific name	Percent identity %	Accession length (bp)	Accession
*Pseudomonas* phage vB_PaeP_FBPa1	94.81	72,814	ON857943.1
*Pseudomonas* phage VB_PaeS_VL1	94.37	73,308	OK665488.1
*Pseudomonas* phage YH6	94.07	73,050	KM974184.1
*Pseudomonas* phage PA26	94.04	72,321	NC_041907.1
*Pseudomonas* phage LP14	94.02	73,080	MH356729.1
*Pseudomonas* phage DL64	94.01	72,378	KR054032.1
*Pseudomonas* phage vB_Pae1396P-5	94.00	72,508	KX171210.1
*Pseudomonas* phage vB_Pae575P-3	94.00	72,728	KX171209.1
*Pseudomonas* phage PAP02	93.97	73,345	MT080102.1
*Pseudomonas* phage vB_PaeP_DEV	93.90	72,697	MF490238.1

### In vivo characterization of the influence of phage vB_PaeP_PS28 on *P. aeruginosa* pathogenesis

**Fig. 9:**
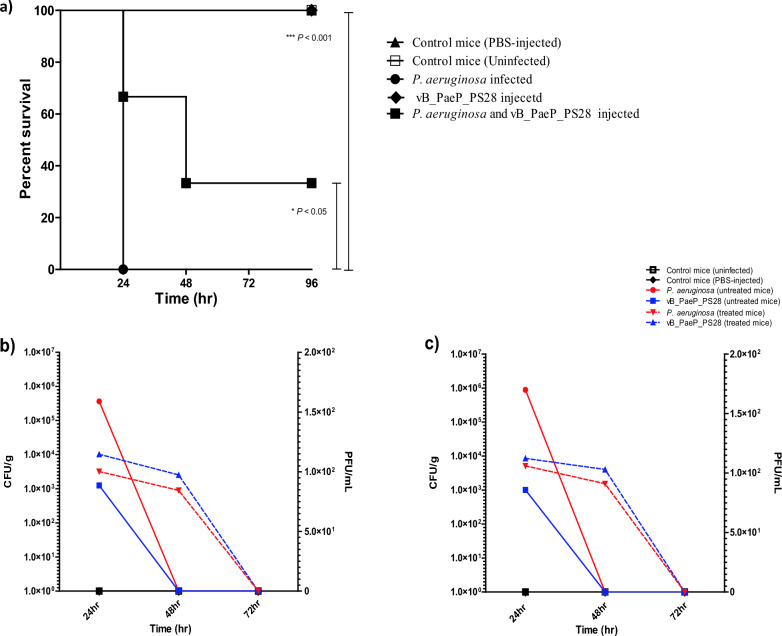
In vivo characterization of the influence of phage vB_PaeP_PS28 on *P. aeruginosa* pathogenesis in mice infection model. **a** Survival curves of mice infected with *P. aeruginosa* and treated with isolated phage. Mice in first group were infected with *P. aeruginosa* (2.5 × 10^7^ CFU/mL), mice in second group were injected with vB_PaeP_PS28 (2.5 × 10^9^ PFU/mL) and mice in third group were infected with *P. aeruginosa* and treated with the phage vB_PaeP_PS28. Uninfected and PBS-injected mice served as negative controls. Mice survival was monitored in each group daily for 4 days and plotted using Kaplan–Meier survival curve. Bacterial burden and phage titers were determined in liver (**b**) and spleen (**c**) of infected mice. Mice were anesthetized, liver and spleen were obtained and homogenized for enumeration of CFU and PFU at 24, 48, 72 h post infection. Of note that, bacterial load in *P. aeruginosa* infected mice was determined at 24 h post infection only as all mice in this group died after 24 h. Bacterial and phage load were represented on left and right y axis, respectively. Data are expressed as means ± SE of three independent experiments

The effect of phage vB_PaeP_PS28 on *P. aeruginosa* pathogenesis was evaluated in vivo using mice infection model. Mice survival as well as both bacterial and phage counts were monitored in infected mice to evaluate whether vB_PaeP_PS28 phage possesses a protective effect in vivo against *P. aeruginosa* virulence. Importantly, phage-injected mice and negative control (non-infected and PBS-injected) mice exhibited 100% survival. On the other hand, all mice infected with *P. aeruginosa* died at 24 h post inoculation. However, the mortality rate of mice infected with *P. aeruginosa* and treated with vB_PaeP_PS28 dramatically decreased as compared to mice infected with bacteria alone **(**Fig. 9a**)**. In addition, bacterial loads were determined in organs isolated from infected mice. As shown in (Fig. 9b, c**)**, the number of viable bacteria in the liver and spleen isolated from mice infected with *P. aeruginosa* and treated with vB_PaeP_PS28 (3193 ± 12, 5134 ± 13 CFUs/g, respectively) was significantly lower than that of mice infected with *P. aeruginosa* alone (36 × 10^4^ ± 23, 88 × 10^4^ ± 15 CFUs/g, respectively). Of note that, the phage titers were determined in isolated organs from phage-injected mice as well as mice infected with *P. aeruginosa* and treated with phage. The phage titer in liver and spleen isolated from *P. aeruginosa* infected mice and treated with vB_PaeP_PS28 was significantly higher (10,241 ± 23, 8520 ± 13, PFU/mL, respectively) as compared to phage injected mice (1250 ± 11, 1005 ± 10, PFU/mL, respectively). Importantly, the phage vB_PaeP_PS28 was rapidly cleared from the liver and spleen isolated from phage-injected mice. Importantly, the phage-injected mice did not develop any abnormal symptoms over the experiment course. These findings clearly demonstrate that vB_PaeP_PS28 phage is promising for therapeutic use and would be cleared from the body without any harmful effects on the patient.

## Discussion

*P. aeruginosa* is responsible for a majority of serious infections including urinary tract and lung infections and pneumonia (Litwin et al. 2021). Due to extensive use of antibiotics and continuous increase of antibiotic resistance, bacteriophages are seemed to be efficient alternatives in management of infections caused by *P. aeruginosa* (Pires et al. 2015). In the current study, a total of 50 *P. aeruginosa* isolates were obtained and screened for their antibiotic susceptibility against different antibiotics. *P. aeruginosa* isolates exhibited a higher resistance towards multiple antibiotics including aminoglycosides, fluoroquinolones and monobactam; revealing that 56% of isolated *P. aeruginosa* strains were MDR. Therefore, phage therapy could be a promising alternative strategy to control the alarming increase in bacterial resistance of *P. aeruginosa*. Bacteriophages are supposed to have several advantages compared to antibiotics as being safer and best tolerated without affecting mammalian cells (Kakasis and Panitsa 2019). Furthermore, there is no need for repeated administration of phage doses which commonly known for antibiotics. There is a significant increase in phage concentration at infection site via auto “dosing” that results in greater bacterial killing following only a single dose (Abedon and Thomas-Abedon 2010).

In the current study, a novel lytic phage vB_PaeP_PS28 infecting *P. aeruginosa* was isolated and characterized. Lytic phages are generally preferred for therapeutic purposes in comparison with temperate phages. Temperate phages exhibit drawbacks including the transfer of virulence genes that could lead to increased antibiotic resistance within bacteria (Monteiro et al. 2019). TEM analysis revealed that vB_PaeP_PS28 is a member of *Podoviridae* family with icosahedral head and short non contractile tail. Tailed phages with double stranded DNA are classified into three different morphological families; *Siphoviridae*, *Podoviridae* and *Myoviridae* within the order *Caudovirales*. *P. aeruginosa* phages have covered all families, and importantly, phages belonging to *Podoviridae* and *Myoviridae* families were found to be of major importance and are highly preferred for phage therapy (Alemayehu et al. 2012; Garbe et al. 2011).

The results of host range and EOP analysis are very important parameters that should be determined when selecting bacteriophages for therapeutic purposes (Viscardi et al. 2008). The phage vB_PaeP_PS28 exhibits a broad host range and was able to lyse 13 out of 18 (72.2%) *P. aeruginosa* strains tested herein. Most of these susceptible strains were highly resistant to traditionally used antibiotics to control *P. aeruginosa* infections including β-lactams (penicillins, carbapenems, cephalosporins, monobactams), aminoglycosides and fluroquinolones. These findings support the effectiveness of isolated phage and its suitability for application in phage therapy. It is well-known that broad host range phages are considered as efficient biocontrol and more preferable for therapeutic application in phage therapy (Fernández et al. 2019). Moreover, the phage vB_PaeP_PS28 fulfills the requirements of pH and temperature stability and showed a higher stability over a wide temperature and pH ranges. Assessment of phages stability under various temperature and pH conditions is critical to provide information regarding phage storage and application. Phages intended for therapy should be stable under drastic conditions to overcome environmental changes during therapeutic applications (Jamal et al. 2017; Fernández et al. 2019). In agreement with the present findings, tailed phages have been shown to be more stable under harsh conditions including temperature and pH changes (Ackermann et al. 2004). These finding are similar to previous studies reporting the isolation of highly stable phages infecting *P. aeruginosa* (Danis-Wlodarczyk et al. 2015). In addition to its stability against various conditions, vB_PaeP_PS28 phage possesses a latent period of 15 min and burst size of 210 virions per infected bacterium. These growth characteristics further support the potential incorporation of phage vB_PaeP_PS28 in treatment of *P. aeruginosa* infections. Phages that have large burst size and short latent period have been reported to be efficient antimicrobial agents (Khan Mirzaei and Nilsson 2015).

Biofilms play an important role in bacterial pathogenesis and could lead to persistence of infections and increased resistance to antibiotics (Mah et al. 2003). Previous reports have demonstrated that bacteriophages are promising in eradicating *P. aeruginosa* biofilms (Tian et al. 2021). Current results indicate a potent antibiofilm activity of vB_PaeP_PS28 against *P. aeruginosa* biofilms confirming its suitability for treatment of *Pseudomonas* infections. The antibiofilm activity of bacteriophages could be related to the production of phage enzymes that degrade polymers in extracellular matrix such as polysaccharides and proteins (Harper et al. 2014). For instance, bacteriophages can encode polysaccharide depolymerase that specifically degrade macromolecular carbohydrates on the host bacterial envelope (Yan et al. 2014). Similarly, bacteriophages produce endolysins which hydrolyse bacterial peptidoglycan, hence inhibit cell wall synthesis (Schmelcher et al. 2012). In addition, bacteriophages could produce enzymes that inhibit quorum sensing in *P. aeruginosa* and therefore suppress biofilm formation (Whiteley et al. 2018). Interestingly, lytic phages have been found to maintain their lytic activity against persister cells within biofilms which are characterized by low metabolic activity (Tian et al. 2021). Therefore, bacteriophages seem to be a suitable option for the fight against persistent biofilms (Fernández-Barat et al. 2012).

Importantly, the genome of vB_PaeP_PS28 phage was sequenced and gene annotation confirmed that phage vB_PaeP_PS28 is a member of *Podoviridae* family and the genus *Litunavirus* of subfamily *Migulavirinae*. The *Litunavirus* is a member of a well-characterized N4-like phage. Almost all of the N4-like phages exhibit highly conserved gene organization and expression (Menon et al. 2021; Wittmann et al 2020). The vB_PaeP_PS28 genome encodes gene (ORF 1) which is highly similar to virion-associated RNA polymerase, a remarkable protein that is characteristic of N4-like viruses and unique among all other phages. N4-like viruses co-inject this enzyme with DNA during infection and is responsible for the transcription of early genes (Farmer et al. 2013). The genomes of the N4-like phages from the *Pseudomonas* group did not reveal any tRNA genes which is in accordance with the annotated vB_PaeP_PS28 phage genome (Wittmann et al 2020). Other genes within phage genome with functional annotation (ORF 8 and ORF 9) have a high percent identity to previously annotated N4-like proteins also supporting the designation of vB_PaeP_PS28 as an N4-like phage. Comparative analysis was performed between vB_PaeP_PS28 phage and other phages infecting *P. aeruginosa* available in the NCBI. The genomic comparison shows that isolated phage has best similarities with previously isolated and characterized phages; *Pseudomonas* phage vB_PaeP_FBPa1 (GenBank Acc. No. ON857943.1), *Pseudomonas* phage vB_PaeS_VL1 (GenBank Acc. No. OK665488.1) and *Pseudomonas* phage YH6 (GenBank Acc. No. KM974184.1) with percent identity of 94.81, 94.37 and 94.07%; respectively. The results were in accordance with the results of VIRIDIC. These findings were further confirmed upon performing a phylogenetic analysis based on the genes encoding for the terminase large subunit and DNA polymerase that are conserved in different classes of bacteriophages (Shapiro and Putonti 2018; Akhwale et al. 2019). These proteins are considered as helpful phylogenetic markers and routinely used in the investigation of several phage groups and describing the phylogenetic positioning of newly isolated phage (Casjens et al. 2005; Wittmann et al. 2014).

Importantly, no tRNA was found in the vB_PaeP_PS28 genome; this suggests that upon entry into the host, the phage vB_PaeP_PS28 is completely dependent on the host tRNA for its translation machinery. A critical aspect related to bacteriophage therapy is the possibility of transduction where bacteriophages could transfer bacterial virulence genes among bacteria leading to increased bacterial resistance (Mahichi et al. 2009; Sillankorva et al. 2010). Importantly, the genome annotation of phage vB_PaeP_PS28 showed the absence of genes related to lysogenic cycle. These findings confirm that phage vB_PaeP_PS28 is a virulent phage and further support the suitability of this phage for therapeutic applications to combat *P. aeruginosa* infections.

In the current study, the efficiency of vB_PaeP_PS28 phage to reduce *Pseudomonas* pathogenesis was assessed in vivo using mice infection model. Intriguingly, there was a significant reduction in the mortality of mice infected with *P. aeruginosa* and treated with the phage vB_PaeP_PS28 as compared to bacteria-inoculated mice without phage treatment. Moreover, phage treatment effectively reduced bacterial colonization in the organs isolated from *Pseudomonas*-infected mice relative to mice injected with bacteria alone. Current results are in accordance with previous in vivo studies reporting similar survival rates in *Pseudomonas*-infected mice following the administration of lytic phages (McVay et al. 2007; Watanabe et al. 2007). Additionally, the phage KPP12 successfully treated *Pseudomonas*-induced keratitis and markedly reduced bacterial load in infected mice (Fukuda et al. 2012). Rezk et al. (2022) reported that topical application of phage ZCPA1 resulted in complete bacterial eradication in skin wounds and led to efficient wound closure (Rezk et al. 2022). These findings clearly suggest that phage vB_PaeP_PS28 could be a promising antibacterial agent against *P. aeruginosa* infections*.*

In conclusion, a lytic phage vB_PaeP_PS28 was isolated belonging to the family *Podoviridae* that targets *P. aeruginosa*. The phage vB_PaeP_PS28 exhibits a broad lytic activity as well as higher stability under various environmental conditions. The phage vB_PaeP_PS28 showed a pronounced inhibitory activity against *P. aeruginosa* planktonic cells as well as a potential antibiofilm activity. The therapeutic efficacy of vB_PaeP_PS28 was investigated in vivo using mice infection model. Treatment with phage vB_PaeP_PS28 markedly reduced mortality in *P. aeruginosa*-infected mice and lowered bacterial colonization in isolated organs. Intriguingly, based on the genome analysis and in vivo findings, the phage vB_PaeP_PS28 could be a novel promising candidate that can be used in controlling of *P. aeruginosa* infections. The phage vB_PaeP_PS28 could be introduced as a biocontrol against *P. aeruginosa* alone or incorporated in phage cocktail therapy. Additionally, the isolated phage could be applied to synergize the action of traditionally used antibiotics targeting *P. aeruginosa* infections.

## Supplementary Information

**Additional file 1: Table S1.** Antibiotic susceptibility of *P. aeruginosa *isolates. **Table S2.** Antibiotic sensitivity and phage susceptibility of *P*. *aeruginosa* isolates from different clinical sources tested for host range determination of vB_PaeP_PS28. **Table S3.** Bacterial and phage count following infection of host and *P*. *aeruginosa* PAO1 with vB_PaeP_PS28. **Fig. S1.** Quantitative evaluation of *P*. *aeruginosa* biofilm formation. **Fig. S2.** Dot Plot comparisons of the genomic nucleotide sequences of vB_PaeP_PS28 and related bacteriophages infecting *P*. *aeruginosa*.

## Data Availability

The authors confirm that the data supporting the findings of this study are available within the article.

## Abbreviations

MH: Muller Hinton
ORFs: Open reading frames
R: Resistant
I: Intermediate
S: Susceptible
TS: Tryptone soya
OD: Optical density
ODc: Cut-off optical density
TEM: Transmission electron microscopy
PFUs: Plaque forming units
CFUs: Colony forming units
MOI: Multiplicity of infection
EOP: Efficiency of plating
MDR: Multidrug resistant
IP: Intraperitoneally
ICTV: International committee taxonomy of viruses
IP: Intraperitoneally
ICTV: International committee taxonomy of viruses
BLAST: Basic local alignment search tool
