# Supplementary material for: Isolation of a bacteriophage targeting Pseudomonas aeruginosa and exhibits a promising in vivo efficacy
Source: AMB Express. 2023 Jul 26;13:79. doi: 10.1186/s13568-023-01582-3 (PMC10371947; doi:10.1186/s13568-023-01582-3)
Supplement: Supplementary file 1 — Additional file 1: Table S1. Antibiotic susceptibility of P. aeruginosa isolates. Table S2. Antibiotic sensitivity and phage susceptibility of P. aeruginosa isolates from different clinical sources tested for host range determination of vB_PaeP_PS28. Table S3. Bacterial and phage count following infection of host and P. aeruginosa PAO1 with vB_PaeP_PS28. Fig. S1. Quantitative evaluation of P. aeruginosa biofilm formation. Fig. S2. Dot Plot comparisons of the genomic nucleotide sequences of vB_PaeP_PS28 and related bacteriophages infecting P. aeruginosa. [file 13568_2023_1582_MOESM1_ESM.pdf]

Additional file information

AMB Express

Isolation of a bacteriophage targeting *Pseudomonas aeruginosa* and exhibits a promising in vivo efficacy

Aliaa Abdelghafar, Amira El-Ganiny, Ghada Shaker and Momen Askoura*

Department of Microbiology and Immunology, Faculty of Pharmacy,
Zagazig University, Zagazig 44519, Egypt

*** Corresponding author:**

Momen Askoura: MMAskoura@pharmacy.zu.edu.eg; momenaskora@yahoo.com

Additional file 1 Table S1: Antibiotic susceptibility of *P. aeruginosa* isolates

Isolate NO	CFP	CAZ	FEP	TZP	PRL	CT	CN	TOB	AK	MEM	CIP	GAT	ATM*
1B**	S	S	S	S	S	S	R	R	R	S	R	R	S
2B	R	R	R	R	R	S	R	R	R	R	R	R	R
3B	R	R	R	R	R	S	R	R	R	R	R	R	R
4B	S	S	S	S	S	S	R	R	R	S	R	R	S
5B	R	R	R	R	R	S	R	R	R	R	R	R	R
6B	S	S	S	S	S	S	S	S	S	S	S	S	S
7B	R	R	R	R	R	S	R	R	R	R	R	R	R
8B	R	R	R	I	R	S	R	R	R	S	R	R	R
9B	R	R	R	R	R	S	R	R	R	R	R	R	R
10B	R	R	R	R	R	S	R	R	R	R	R	R	R
11W	S	S	S	S	S	S	S	S	S	S	S	S	S
12W	R	I	R	I	R	S	R	R	R	R	R	R	S
13W	R	R	R	R	R	S	R	I	R	R	R	R	I
14W	S	S	I	S	S	S	I	S	I	I	S	S	I
15W	S	S	S	S	S	S	S	S	S	S	S	S	S
16W	R	R	R	R	R	S	R	R	R	R	R	R	S
17W	R	R	R	I	I	S	R	R	R	R	R	R	S
18W	R	S	I	I	R	S	R	R	S	S	S	S	S
19W	R	S	I	I	R	S	R	R	S	S	S	S	S
20W	S	S	S	S	S	S	I	S	S	I	S	S	S
21U	R	S	R	R	R	S	R	R	R	R	R	R	S
22U	R	R	R	I	I	S	R	R	R	R	R	R	S
23U	R	S	R	R	R	S	R	R	R	R	R	R	S
24U	R	S	R	R	R	S	R	R	R	R	R	R	S
25U	S	S	S	S	S	S	S	S	S	S	S	S	S
26U	S	S	S	S	S	S	S	S	S	S	S	S	S
27U	R	R	R	R	I	S	R	R	R	R	R	R	S
28U	R	R	R	R	R	S	R	R	R	R	R	R	S
29U	R	S	R	R	R	S	R	R	R	R	R	R	R
30U	S	S	I	S	S	S	R	R	R	R	R	R	S
31U	R	R	R	I	I	S	R	R	R	R	R	R	I
32SP	R	R	R	R	R	S	R	R	R	R	R	R	S
33SP	S	S	S	S	S	R	S	S	S	S	S	S	S
34SP	S	S	S	S	S	S	S	S	S	S	S	S	S
35SP	R	R	R	R	R	S	R	R	R	R	R	R	S
36SP	S	S	S	S	S	S	S	S	S	S	S	S	S
37SP	S	S	S	S	S	S	S	S	S	S	S	S	S
38SP	S	S	S	S	S	S	S	S	S	S	S	S	S
39SP	S	S	S	S	S	S	S	S	S	S	S	S	S
40SP	R	R	R	I	I	S	R	R	R	R	R	R	S
41SP	S	S	S	S	S	S	S	S	S	S	S	S	S
42SP	R	R	R	R	R	S	R	R	R	R	R	R	S
43SP	S	S	S	S	S	S	S	S	S	S	S	S	S
44SP	R	R	R	R	R	S	R	R	R	R	R	R	R

**45SP**	S	S	S	S	R	S	S	S	S	S	S	S	S
**46SP**	R	R	R	R	R	S	R	R	R	R	R	R	S
**47SP**	S	S	S	S	S	S	S	S	S	S	S	S	S
**48SP**	I	S	S	S	I	S	S	S	S	R	S	S	S
**49E**	S	S	S	S	S	S	S	S	S	R	S	S	S
**50E**	S	S	S	S	S	S	I	S	S	I	S	S	S

[*]: Amikacin (AK, 30µg), gentamycin (CN, 10µg), ciprofloxacin (CIP, 5µg), meropenem (MEM, 10µg), pipracillin (PRL, 100µg), cefoperazone (CFP, 75µg), piperacillin/tazobactam (TPZ, 110µg), cefepime (FEP, 30µg), ceftazidime (CAZ, 30µg), aztreonam (ATM, 30µg), colistin (CT, 10µg), tobramycin (TOB, 10µg) and gatifloxacin (GAT, 5µg).

[**]: Burn (B); surgical wound (W); urine (U); endotracheal aspirate (SP); ear (E)

Additional file 1 Table S2: Antibiotic sensitivity and phage susceptibility of *P. aeruginosa* isolates from different clinical sources tested for host range determination of vB_PaeP_PS28

Isolate	Source	Antibiotic sensitivity	Phage susceptibility*
**PS 3B**	Burn	MDR**	+
**PS 6B**	Burn	S***	+
**PS 9B**	Burn	MDR	-
**PS 10B**	Burn	MDR	+
**PS 11W**	Wound	S	-
**PS 13W**	Wound	MDR	+
**PS 14W**	Wound	S	+
**PS 22U**	Urine	MDR	+
**PS 23U**	Urine	MDR	+
**PS 24U**	Urine	MDR	+
**PS 28U**	Urine	MDR	+
**PS 32SP**	Endotracheal aspirates	MDR	+
**PS 38SP**	Endotracheal aspirates	S	+
**PS 41SP**	Endotracheal aspirates	S	-
**PS 49E**	Ear infection	S	-

*** Phage susceptibility;** (+) Susceptible, (-) Unsusceptible, ****MDR;** Multi-drug resistant (resistant to at least one antibiotic from three or more antimicrobial classes), *****S;** Sensitive.

Additional file 1 Table S3: Bacterial and phage count following infection of host and *P. aeruginosa* PAO1 with vB_PaeP_PS28

**Bacterial strain/MOI**	**Bacterial count (CFU/mL)**		**Phage count (PFU/mL)**	
	**PS28 (host)**	***Pseudomonas aeruginosa* PAO1**	**PS28 (host)**	***Pseudomonas aeruginosa* PAO1**
**Control (Untreated)**	$2.5 \times 10^9 \pm 2.7 \times 10^2$	$5 \times 10^9 \pm 2.5 \times 10^2$	-	-
**MOI = 10**	$1.5 \times 10^7 \pm 1.6 \times 10^2$	$1.7 \times 10^7 \pm 2.4 \times 10^2$	$4.5 \times 10^{16} \pm 5.4 \times 10^2$	$2 \times 10^{14} \pm 3.8 \times 10^2$
**MOI = 1**	$2 \times 10^7 \pm 1.62 \times 10^2$	$2.1 \times 10^7 \pm 2.57 \times 10^2$	$2.5 \times 10^{15} \pm 4 \times 10^2$	$5 \times 10^{12} \pm 3.2 \times 10^2$
**MOI = 0.1**	$3.1 \times 10^7 \pm 1.73 \times 10^2$	$2.9 \times 10^7 \pm 2.53 \times 10^2$	$2.9 \times 10^{15} \pm 4.2 \times 10^2$	$2.5 \times 10^{11} \pm 3 \times 10^2$

Additional file figures

Additional file 1 Fig. S1

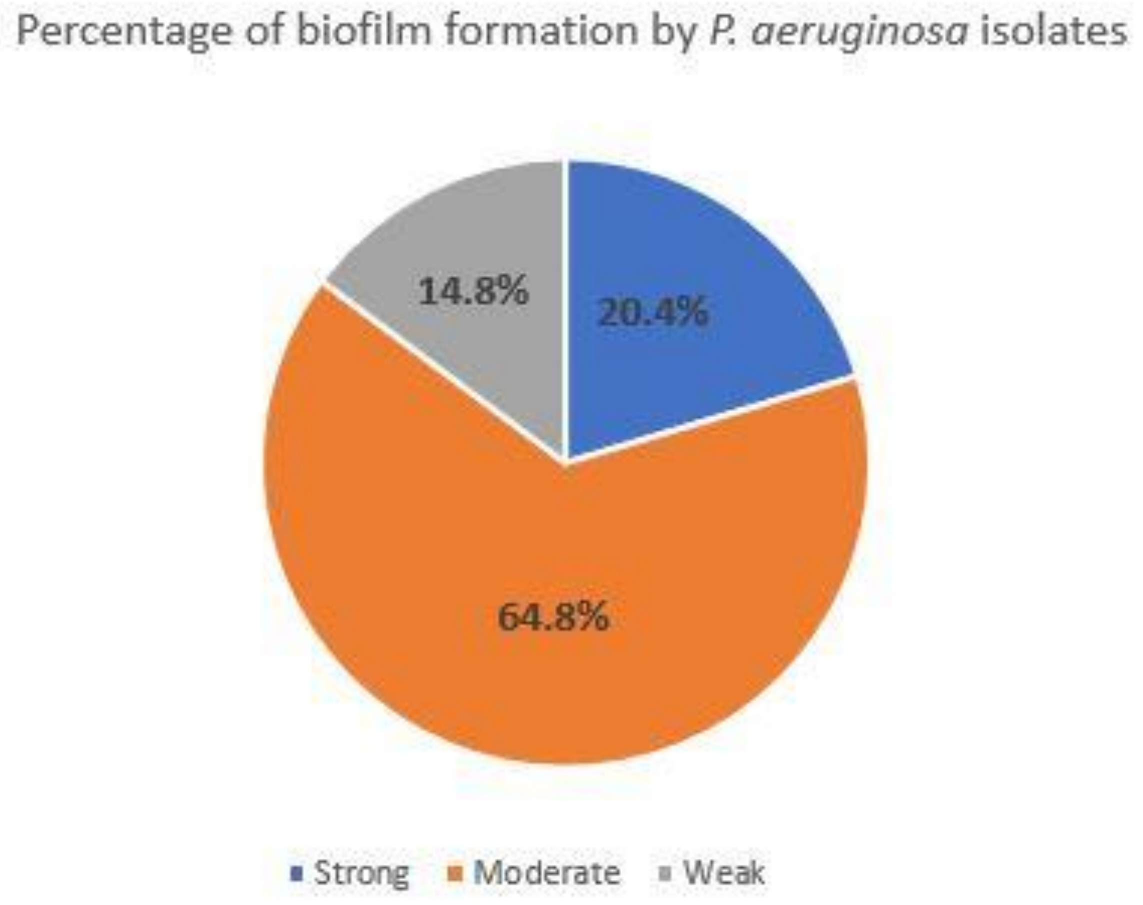

Additional file 1 Fig. S2

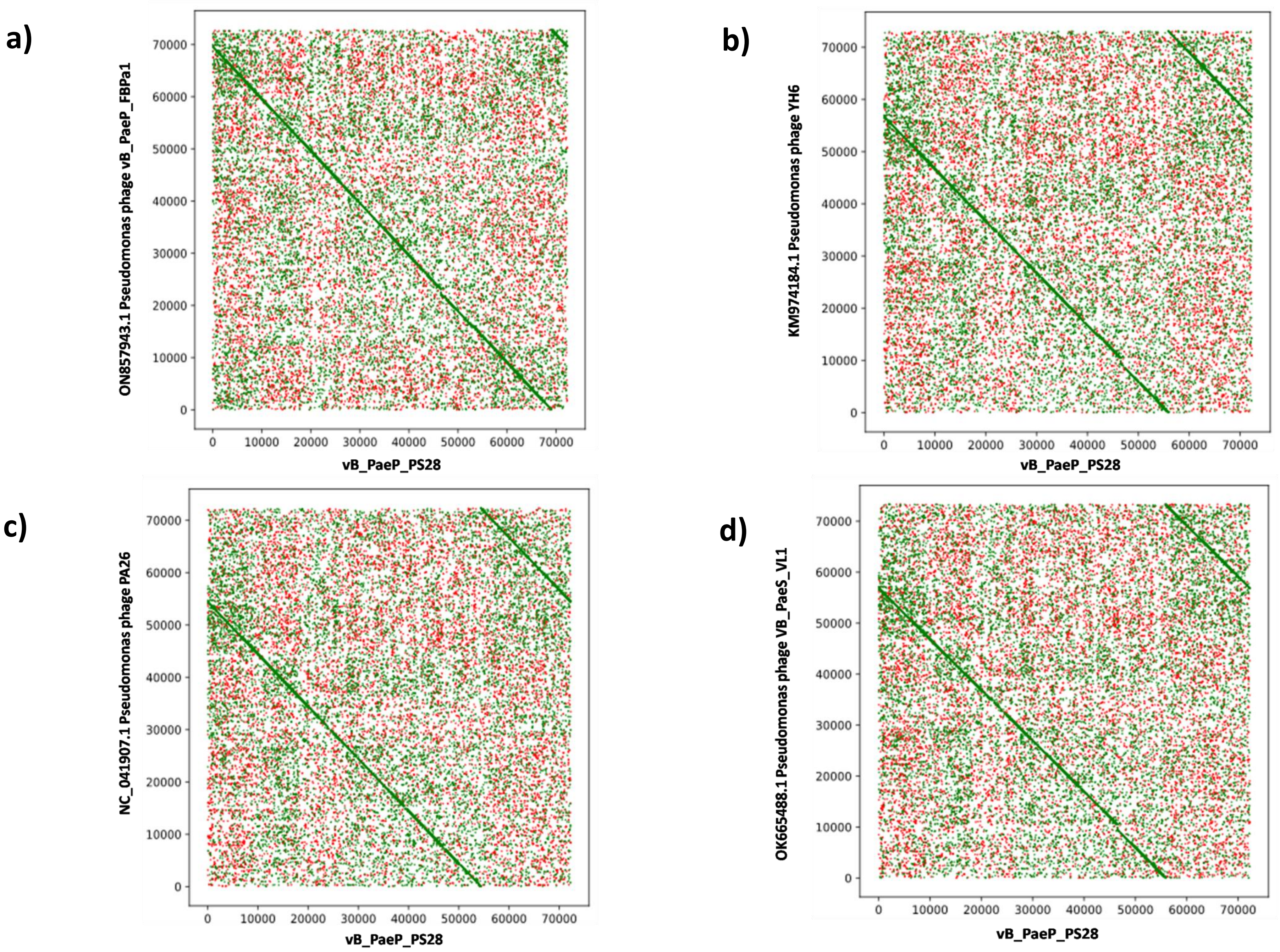

Supplementary figure legends

Supplementary Fig. S1 Quantitative evaluation of *P. aeruginosa* biofilm formation. Bacterial biofilms were stained with crystal violet (CV), solubilized by 33% glacial acetic acid and measured spectrophotometrically at OD_{570}. *P. aeruginosa* isolates were categorized into strong, moderate and weak biofilm forming

Supplementary Fig. S2 Dot Plot comparisons of the genomic nucleotide sequences of vB_PaeP_PS28 and related bacteriophages infecting *P. aeruginosa*. a) *Pseudomonas* phage vB_PaeP_FBPa1 (GenBank Acc. No. ON857943.1). **b)** *Pseudomonas* phage YH6 (GenBank Acc. No. KM974184.1). **c)** *Pseudomonas* phage PA26 (GenBank Acc. No. NC_041907.1). **d)** *Pseudomonas* phage VB_PaeS_VL1 (GenBank Acc. No. OK665488.1
